# The Measurement and Conceptualization of Coping Responses in Pediatric Chronic Pain Populations: A Scoping Review

**DOI:** 10.3389/fpsyg.2021.680277

**Published:** 2021-10-01

**Authors:** A. Natisha Nabbijohn, Rachel M. Tomlinson, Soeun Lee, Barbara A. Morrongiello, C. Meghan McMurtry

**Affiliations:** ^1^Department of Psychology, University of Guelph, Guelph, ON, Canada; ^2^Pediatric Chronic Pain Program, McMaster Children's Hospital, Hamilton, ON, Canada; ^3^Children's Health Research Institute and Department of Paediatrics, Schulich School of Medicine and Dentistry, London, ON, Canada

**Keywords:** coping, coping responses, pediatric chronic pain, scoping review, conceptualization and measurement

## Abstract

**Background:** Pediatric chronic pain is a prevalent condition that requires significant coping to encourage optimal functioning; however, relevant research is vast, heterogeneous, and difficult to interpret. To date, no attempt has been made to map and summarize the measurement and conceptualization of coping responses in the context of pediatric chronic pain.

**Objectives:** A scoping review was conducted to map and summarize the participant characteristics, methodologies, theoretical frameworks, and measures used to assess coping responses in youth with chronic pain. The extent to which authors used definitions and examples of coping responses (conceptual clarity) as well as consistently used measures (measurement consistency) and their corresponding conceptualizations (conceptual consistency) relative to how they were intended to be used were assessed.

**Methods:** Searches were conducted through MEDLINE (PubMed) and PsycINFO. Following title/abstract screening, full-text extractions were performed on 125 English-language publications on coping in youth with chronic pain.

**Results:** Of the 125 studies, only 12.8% used a theoretical framework to explain the coping responses assessed, and even fewer (7.2%) used theory to guide measure selection. Conceptual clarity was rated “low/very low” (i.e., no definitions and/or examples) for 47.2% of studies. The majority of studies were conducted in the United States (67%) and a preponderance of White and female participants was sampled. The research primarily used quantitative methods (85%) and cross-sectional designs (67%). Parent- or self-report questionnaires were the most common methods for assessing coping (86%). Of the 95 studies that utilized one of the 14 questionnaires with known psychometric properties, 33.7 and 55.8% had one or more discrepancies for conceptual and measurement consistency, respectively.

**Conclusions:** This review highlights the lack of clear descriptions and theoretical frameworks of coping responses for pediatric chronic pain. Inconsistencies in the measurement and conceptualization of coping responses limit research and clinical advancements. As a field, we need to strive toward using well-developed theory to create fewer, more well-established standardized measures with clearly defined coping responses. Opportunities for qualitative and observational research in more diverse patient populations should be considered for theory construction and measure validation.

**Clinical Trial Registration:** https://osf.io/xvn2a/?view_only=eff04e0c0b9649be89d403b10e9ff082.

## Introduction

Pediatric chronic pain refers to persistent or recurrent pain in infants, children, and adolescents (herein “youth”) that lasts for more than 3 months and lacks an adaptive purpose (Treede et al., 2015). Given the long-lasting and unpredictable nature of chronic pain, youth rely on coping to manage their pain and its impact (Peres and Lucchetti, 2010). Coping is defined as the use of intentional and effortful thoughts or behaviors to manage the internal and external demands of stressful situations or experiences (Compas et al., 2014). Rudolph et al. (1995) proposed a model that conceptualizes coping to occur in a sequence of events (i.e., a “coping episode”) consisting of coping responses, goals, and outcomes. Within this model, coping responses are defined as mental or physical actions initiated in relation to a perceived stressor (Rudolph et al., 1995). Coping goals are the reasons for engaging in a particular coping response, and coping outcomes are the specific consequences of a coping response. Coping responses may serve different goals and be related to different outcomes across time and situations (Skinner et al., 2003). Therefore, our ability to understand the efficacy and implementation of a coping episode depends on successfully identifying and measuring coping responses. Although there are other models of coping (Skinner et al., 2003; Stanisławski, 2019), this review uses the abovementioned terminology to summarize the literature.

The coping literature is vast, heterogenous, and difficult to interpret. In an effort to better understand this literature, reviews have summarized and critically evaluated the measurement and conceptualization of coping in adult chronic pain (Peres and Lucchetti, 2010), childhood chronic illness (Rudolph et al., 1995; Compas et al., 2012), and general stress (Jensen et al., 1991; Skinner et al., 2003; Garcia, 2010; Stanisławski, 2019); however, there is no review of coping in pediatric chronic pain. This is a critical gap considering that coping responses may vary by health condition as well as age (Garcia, 2010; Compas et al., 2012). For example, chronic pain is often perceived as less predictable and controllable than other chronic illnesses (Compas et al., 2012). Consequently, youth with chronic pain may rely more on coping responses aimed at *adapting* to their pain such as “distraction” or “acceptance” rather than efforts to *change* their pain or emotional response to the pain (e.g., “problem-solving”, “emotional regulation”). Moreover, developmental changes in perceived control and competence, in combination with sociocultural factors (e.g., culture and gender), influence the way people choose and implement various coping responses (Compas et al., 1991; Compas, 1998). For instance, children and young adolescents may be less capable of managing their emotional reactions to their pain and depend more directly on their caregivers to cope than older adolescents and adults (Skinner and Zimmer-Gembeck, 2016). Therefore, a comprehensive review of the existing literature on coping in the context of pediatric chronic pain is needed to identify and evaluate measures and conceptualizations of coping responses specific to this population.

In the broader coping literature, several important gaps limit the ability to communicate effectively about coping and consolidate research on effectiveness. With regard to conceptual gaps, there is a lack of consensus about how best to classify and define coping responses (Skinner et al., 2003; Stanisławski, 2019). As such, coping has been conceptualized using over 400 coping responses organized within more than 100 different typologies (Skinner et al., 2003). Typologies of coping are generally hierarchical and multidimensional organization systems where specific coping responses are unidimensional lower-order categories nested within more complex and abstract higher-order categories of coping. Correspondingly, the most widely used conceptualizations of lower- and higher-order coping responses in the pediatric coping literature include coping *strategies* (i.e., specific and discrete cognitive, emotional, and/or behavioral responses, such as “planning” or “distraction”) and coping *styles* (i.e., a set of coping strategies that fulfill a specific function showing relative stability over time and situations, such as “problem-focused” or “emotion-focused” coping), respectively (Stanisławski, 2019). The extent to which other terminologies have been used remains unclear. Furthermore, the organization of lower-order into higher-order categories is typically accomplished according to their intended function (i.e., the coping goal) and topological distinction (i.e., descriptive categories that are concerned with *how* children cope) (Skinner et al., 2003). However, given that some lower-order coping responses include multiple functions and behaviors, the use of a classification system may contribute to inconsistencies across coping measures. For example, within “problem-focused” coping (i.e., responses aimed at modifying or eliminating the stressor) and “emotion-focused” coping (i.e., responses aimed at managing the emotions aroused by the stressor), the response of “planning” (identified as a lower-order category on scales assessing coping) can be functional for both “problem-focused” and “emotion-focused” coping by guiding problem-solving and calming negative emotions, respectively. Although theories and definitions can guide more consistent conceptualizations of coping responses, they are seldom used (Garcia, 2010). Addressing conceptual inconsistencies (i.e., varied terminologies/categorizations) and ambiguities is critical as they limit our ability to draw conclusions from research and make real-world applications.

Existing reviews also highlight the limitations of having too many measures of coping. Within the general pediatric coping literature, at least 52 measures of a child coping have been identified (i.e., 38 self-report measures, eight observational measures, and six caregiver-report measures) (Blount et al., 2008). The existence of a large number of measures that vary in content and structure contributes to an excessive number of coping responses and makes it difficult to compare and consolidate research findings across studies. In addition, the lack of clear theories and descriptions of coping responses makes it difficult to understand the similarities and differences across measures.

The *timing* in which coping is assessed in relation to the identified stressor is also not well-understood. Within pediatric chronic pain, proactive coping would include efforts or goals undertaken in advance of a painful episode to prevent it or reduce its severity (e.g., practicing daily mindfulness), and reactive coping would include responses to experiencing pain (e.g., listening to music to distract from pain). Distinguishing between proactive and reactive coping is particularly important for intervention planning. For instance, assessments of proactive coping may be more relevant to interventions aimed at facilitating lifestyle changes. In contrast, interventions aimed at helping youth learn how to cope in response to pain may require assessments of what coping responses are used during painful situations (Ho, 2019). The extent to which studies have appropriately considered the timing of coping in response to pain in the selection and interpretation of coping measures has not yet been examined.

**Figure 1:**
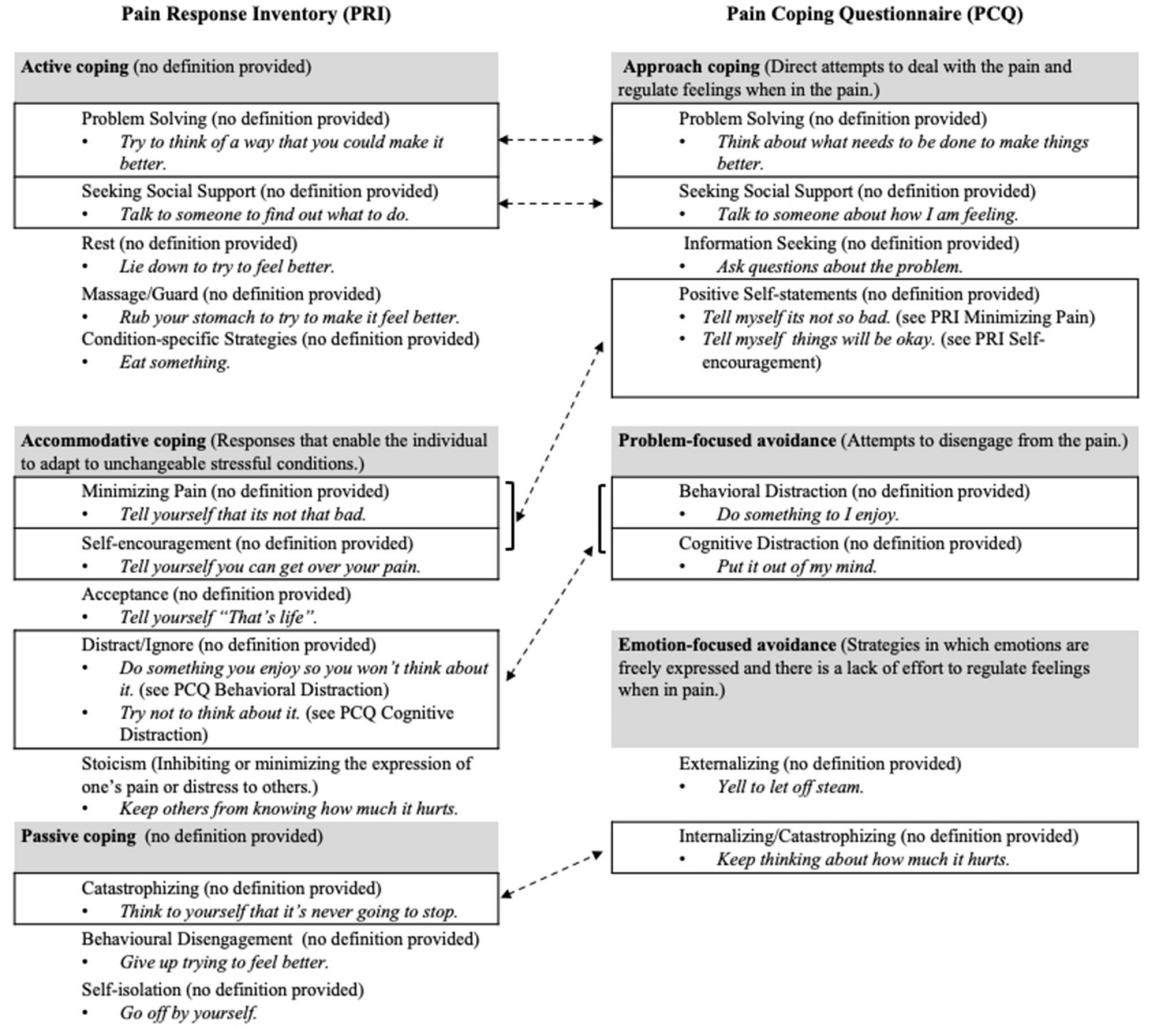
Using the PRI (Walker et al., 1997) and the PCQ (Reid et al., 1998), this figure illustrates the inconsistencies in content (e.g., items, subscales) and organization (categorizations of coping responses) as well as the use of definitions across measures. The gray boxes indicate the higher-order factors, and the lined boxes indicate subscales that appear conceptually similar across measures. Example items are provided in italics. Dotted lines connect subscales that appear to be similar on each measure and demonstrate differences in their categorization within higher-order categories. Of note, the PRI and PCQ assess three different higher-order factors. Some lower-order coping responses either sound the same (e.g., “problem-solving”) or consist of similar items (e.g., “positive self-statements”, “minimizing pain”, and “self-encouragement”), but the extent to which these seemingly similar subscales should be conceptualized the same way is unclear due to the lack of clear definitions and inconsistent categorizations within higher-order factors [i.e., “problem solving” as “active coping” (PRI) vs. “approach coping” (PCQ); “seeking social support” as “active coping” (PRI) vs. “approach coping” (PCQ); “catastrophizing” as “passive coping” (PRI) vs. “emotion-focused avoidance” (PCQ); “positive self-statements” (conceptually similar to “self-encouragement” and “minimizing pain” combined) as “accommodative coping” (PRI) vs. “approach coping” (PCQ); and “distract/ignore” (conceptually similar to “cognitive distraction” and “behavioral distraction” combined) as “accommodative coping” (PRI) vs. “problem-focused avoidance” (PCQ)].

In sum, our ability to perform research and effectively communicate about how youth cope with pain is limited by: (i) the vastness of the literature; (ii) the use of unclear and inconsistent terminologies and categorizations of coping responses; and (iii) inconsistencies in how coping responses are measured across studies. Figure 1 illustrates content, organizational, and definitional challenges by comparing the subscales of the pain response inventory (PRI) (Walker et al., 1997) and the pain coping inventory (PCQ) (Reid et al., 1998), the two most frequently cited (Web of Science citation count: PRI = 197 articles and PCQ = 164) and well-established measures of pain-related coping in youth (Blount et al., 2008). The extent to which these challenges apply in the field of pediatric chronic pain remains unclear. Thus, a scoping review was conducted to systematically map the measurement and conceptualization of coping in populations with pediatric chronic pain. Specific research questions were as follows:

1) *In whom, what, why, and how are coping responses measured*? Specific characteristics of interest were: (i) The “who”—the sample characteristics (i.e., gender, age, ethnicity, and pain conditions) and study characteristics (i.e., country, year); (ii) the “what”—research methodologies employed (i.e., study design, type of data); (iii) the “why”—applications of theoretical frameworks by study authors; and (iv) the “how”—types of coping measures used and their characteristics (i.e., the name of the measure, purpose, response options, internal reliability of subscales, assessment of proactive vs. reactive coping, parent or youth report, the types of coping responses captured by the subscales, and coping structure used).
2) *How have coping responses been conceptualized in the literature*? This was examined based on: (i) the operationalized definitions and/or descriptions of coping responses used; (ii) the clarity with which authors defined or described the coping responses measured; (iii) the terminology used to classify coping responses within higher- and lower-order categories (e.g., coping strategies vs. coping styles); and (iv) the extent to which the selection of coping measures was grounded in a clear theoretical or empirical rationale (herein “concept guided”).
3) *Are coping responses measured and conceptualized consistently relative to their intended purpose*? This review evaluated the extent to which: (i) the appropriate validation studies were cited; (ii) measures were used consistently (i.e., measurement consistency); and (iii) concepts were described consistently with what the original scale purports to measure (i.e., conceptual consistency).

## Methods

This scoping review was developed using the methodological framework put forward by Arksey and O'Malley (2005) and further refined by the Joanna Briggs Institute (Peters et al., 2019) and was written in accordance with the PRISMA-ScR reporting guidelines (Tricco et al., 2018). A scoping review approach allows for a descriptive account of the available information in a particular field and is ideal for broadly defined research questions and heterogeneous sources of evidence, which can then guide future narrower systematic reviews (Sucharew, 2019). The literature reviewed was derived from a larger review of positive psychological factors in the context of pediatric chronic/recurrent pain (see Supplementary Materials); only peer-reviewed articles pertaining to youth pain-related coping were included in this study. The study protocol and materials are available *via* Open Science Framework (https://osf.io/xvn2a/?view_only=eff04e0c0b9649be89d403b10e9ff082).

### Eligibility Criteria

#### Population

Studies that examined a pediatric population (youth 0–18 years of age). Studies with participants above the age of 18 were retained only if they included a pediatric sample that extended into young adulthood with a maximum age of 21 (Hardin and Hackell, 2017). Studies that examined adult, both pediatric and adult (above 21 years of age), and/or animal populations were excluded.

#### Concept

The main concept of interest is youth pain-related coping, which includes the use of coping responses by youth to manage pain or pain-related stressors. Pain catastrophizing in the context of coping (i.e., the tendency to magnify the threat associated with pain and to forecast negative outcomes) (Quartana et al., 2009) was excluded because it has been well-documented as a non-adaptive cognitive–affective response to chronic pain that has been reviewed on its own (Vervoort et al., 2006; Leung, 2012). The exclusion of catastrophizing is also consistent with previous reviews, which have conceptualized catastrophizing as a unique psychological factor rather than a type of coping response (Sharma et al., 2020).

#### Context

Studies including youth with chronic pain conditions (i.e., persistent and/or recurrent pain) were included. Examples of included pediatric chronic pain conditions are sickle cell disease, juvenile arthritis, complex regional pain syndrome, and abdominal pain, among others. In line with other pediatric chronic pain reviews (Lewandowski et al., 2010; King et al., 2011; Eccleston et al., 2014), studies that exclusively examined a diverse pediatric chronic illness group (e.g., non-chronic pain disorders such as diabetes and asthma) or cancer-related pain were excluded. Finally, studies that examined “recurrent pain” in healthy samples drawn from community or school samples of children were excluded.

#### Types of Evidence Sources

English language articles containing original data published in scientific journals were included. All non-peer-reviewed publications (e.g., dissertations), reviews, commentaries, editorials, and chapters were excluded. Non-English language studies were excluded due to feasibility (i.e., the lack of translators on the research team).

### Stage One: Database Searches and Screenings

Electronic literature searches were conducted by a lead researcher (RMT or ANN) in MEDLINE (PubMed) and PsycINFO in three phases between March 26, 2015 and August 20, 2020. Databases were selected in accordance with Cochrane Review recommendations for reviews in the fields of medicine and psychology (Higgins and Green, 2008). All citations were uploaded into EndNote, and the titles and abstracts were screened for inclusion prior to conducting full-text screenings and extractions. Upon completion of a training period (i.e., 100 articles), title/abstract screenings were completed by two reviewers within EndNote, including undergraduate research assistants and/or a lead researcher (RMT or ANN), and inter-rater reliability was calculated based on percentage agreements.

The first two phases (March 26, 2015; September 4, 2018) employed double coding for 20% of all articles identified and had inter-rater reliability of 89% at the abstract level. In the third phase (August 20, 2020), all title/abstract screenings were double-coded and had an inter-rater agreement of 98.3%. Articles were then screened at the full-text level by two reviewers. All included articles were checked by the lead researcher (ANN) prior to inclusion, and any discrepancies were resolved through discussion.

### Stage Two: Data Extraction

Information was extracted on study details (e.g., the year of study, country), participant characteristics (e.g., gender, age), and methodological characteristics (e.g., study designs, types of data collected). In addition, characteristics of the measures used (e.g., number of items, the content/structure, timing of coping assessed) and the terminologies and conceptualizations of the coping responses assessed (e.g., theoretical frameworks, definitions/descriptions, categorization of coping types) were extracted from the included studies. The appropriateness of the measure selected in each included study of the review was evaluated based on whether the concepts assessed were in alignment with the stated research objectives/hypotheses and/or theoretical framework of the authors (i.e., a concept-guided approach), which is a recommended approach for developing and validating knowledge (Coster, 2013; Boateng et al., 2018). In order to map the current published research literature, study authors were not contacted for missing information.

A standardized data extraction form was piloted on a random sample of 10 included articles and modified as required based on feedback from other reviewers. A final revision and the pilot of the extraction spreadsheet were completed on July 10, 2020. All extractions were completed by the primary researcher (ANN) and were reviewed and verified by a second reviewer (an undergraduate research assistant). The number of disagreements between reviewers per article ranged from 0 to 4 out of 45 decisions made per article (*Mdn* = 1.00, *M* = 0.99, *SD* = 1.08) and was resolved through discussion between the two reviewers or adjudication by a third researcher (CMM) as needed. The following subsections will outline key areas of consideration and procedures used in the data extraction process.

### Stage Three: Comparisons With Original Scale Development Studies

Scale development/validation studies were identified from the database search (*n* = 4) as well as *via* snowball searching (i.e., using the included studies as a starting point and pursuing references for assessment tools cited by the study authors; *n* = 20). Appropriateness of the measure selected, as well as the accuracy and clarity of the use of the coping measures and concepts, was determined by examining information from the scale development studies. An “overview document” (see Supplementary Materials for a sample) of the scale development studies was developed for each measure that included information on the participant characteristics, theoretical background, measure characteristics, and definitions of coping responses. Each overview document was checked by a lead researcher (ANN) prior to being used.

For studies that used a questionnaire with published information on its psychometric properties, comparisons between each included study and the respective scale development study/studies were completed to determine measurement consistency, conceptual consistency, and conceptual clarity (see Table 1 for more information). For conceptual clarity and consistency, the use of both definitions (i.e., a statement that describes the meaning of a concept) and examples (i.e., describing a specific behavior or naming subtypes of coping responses) were evaluated because they are both important elements of a well-established concept (Gerring, 1999) and have been used to explain coping in previous research (Garcia, 2010). The rating scales employed were adapted from recommendations by the Cochrane Review Groups to maximize the simplicity and clarity of the coding schemes (Lundh and Gøtzsche, 2008). These rating scales are not an assessment of methodological quality but rather highlight when our ability to directly interpret, consolidate, and/or compare research findings may be limited. All three rating scales were evaluated by two reviewers (an undergraduate research assistant and ANN), and agreement was assessed using percent agreement. High agreement was found between reviewers for ratings of conceptual clarity (91.2%), measurement consistency (88.4%), and conceptual consistency (89.5%).

**Table 1:** Operationalization of measurement consistency, conceptual consistency, and conceptual clarity (adapted from the Cochrane Review Groups recommendations) (Lundh and Gøtzsche, 2008).

**Domain**	**Description**	**^a^Ratings**
Measurement consistency	The extent to which the author appropriately used the measure selected.	**High:** The use of the coping measure employed was fully consistent with the scale development study. **Low:** There is at least one discrepancy in how the coping measure was used relative to the scale development study. **Unclear:** Unable to evaluate measurement consistency due to insufficient information (e.g., missing more than two relevant characteristics)
Conceptual consistency	The extent to which the ^b^descriptors of coping used in a particular study are consistent with the descriptors that were proposed by the scale development study.	**High:** The descriptors of all coping constructs were fully consistent with the scale development study. **Low:** There is at least one discrepancy in how coping was described between the study and scale development study. **Unclear:** Unable to evaluate conceptual consistency due to insufficient information (e.g., there are no descriptors of the coping constructs in the study or the corresponding scale development study).
Conceptual clarity	The extent to which coping constructs were defined or described using examples.	**High:** All relevant coping terms were clearly defined AND potential applications of the coping construct were provided. **Moderate:** All relevant coping terms were either clearly defined OR potential applications of the coping construct were provided. **Low:** Some relevant coping terms were defined and/or potential applications were provided. **Very Low:** No relevant coping terms were defined, and no potential applications were provided.

a *Ratings are used to indicate the presence of discrepancies that may impact our ability to directly interpret, consolidate, and/or compare research findings. These ratings do not reflect an assessment of the quality of the research*.

b *Descriptors = refer to the use of definitions and/or examples*.

### Data Synthesis

The results were summarized using a combination of descriptive numerical and narrative summaries in accordance with the research questions. All numerical descriptive statistics (means and proportions) were conducted using SPSS version 26. Weighted means were used to summarize participant characteristics (i.e., age and gender) to account for sample size differences.

## Results

**Figure 2:**
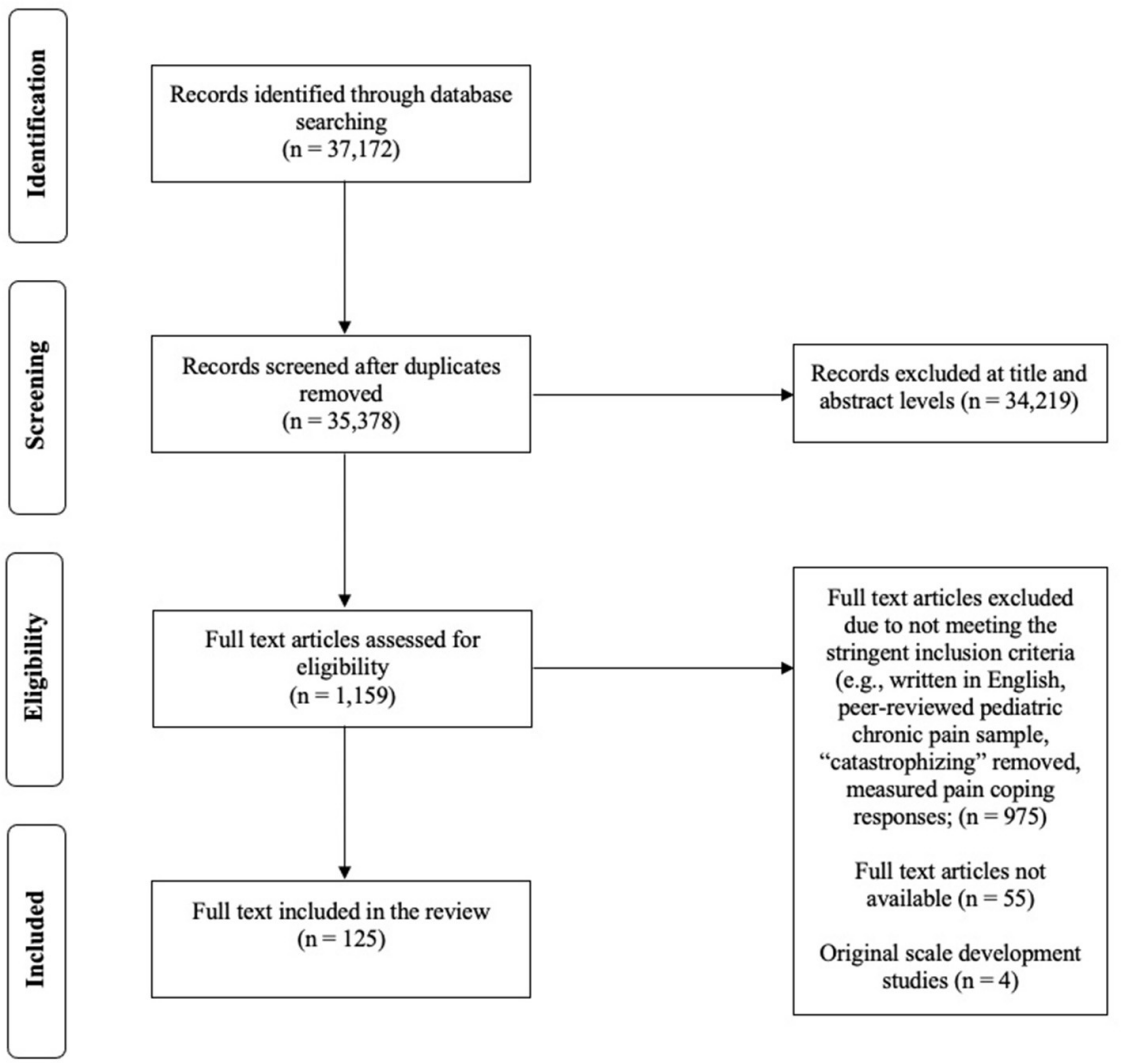
Included a study flow chart following PRISMA guidelines.

As shown in Figure 2, database searching identified 37,172 potential articles encompassing a wider range of positive psychological factors associated with adjustment in youth with chronic pain. After the removal of duplicates and articles that did not meet the inclusion criteria for this review at the title/abstract level of screening, 1,159 articles remained. Of these articles, 129 articles met the eligibility criteria for full-text review. Scale development/validation studies identified from the database search (*n* = 4) were reviewed separately and used as a reference to evaluate the measurement and conceptual consistency of research studies in the field. Thus, 125 peer-reviewed articles related to coping and pediatric chronic pain were included in this review (see Supplementary Table 1 for a list of included studies) (Moher et al., 2009).

### Aim 1: The Who, What, Why, and How of Measuring Pain-Related Coping in Youth

Table 2 provides an overview of the included studies; see Supplementary Table 2 for the participant, study, and methodological characteristics of each individual study. Studies were published between 1991 and 2020. The vast majority of studies (88%) were conducted in the USA (*n* = 84; 67.2%) and countries in Western Europe (*n* = 26; 20.8%).

**Table 2:** Overview of characteristics of the included studies (*N* = 125).

**Characteristics**		**No. of studies (%)**	**Characteristics (cont.)**		**No. of studies (%)**
*Geographic region*	USA	84 (67.2)	*Predominant ethnicity/race (>50% of sample)*	Caucasian	41 (32.8)
	^a^Europe	26 (20.8)		^d^African American	32 (27.2)
	Canada	7 (5.6)		^e^Other	3 (2.4)
	Australia	3 (2.4)		Not reported	47 (37.6)
	^b^Rest of the world	5 (4.0)			
			*Methods*	Quantitative	108 (84.6)
*Year*	1991–1995	12 (9.6)		Qualitative	13 (10.4)
	1996–2000	14 (11.2)		Mixed	4 (3.2)
	2001–2005	19 (15.2)			
	2006–2010	28 (22.4)	*Informant*	Child	93 (75.2)
	2011–2015	24 (19.2)		Parent	5 (4.0)
	2016–2020	28 (22.4)		Multi-informant	21 (16.8)
				Behavioral	1 (0.8)
*Sample size*	<50	52 (41.6)		Not specified	5 (4.0)
	50–100	33 (26.4)			
	101–500	37 (29.6)	*Design*	Case study/series	2 (1.6)
	501–1,000	3 (2.4)		Cross-sectional	84 (67.2)
				Longitudinal	24 (19.2)
*Age-range*	Children	10 (8.0)		RCT	12 (9.6)
	(<12 years)			^f^Other	3 (2.4)
	Adolescent	28 (22.4)	*Control group*		
	(>12 years)			Chronic pain	23 (18.4)
	Both	85 (68.0)		Non-chronic pain	11 (8.8)
	Not specified	2 (1.6)		None	91 (72.8)
*Pain condition*	SCD	42 (33.6)	*Intervention used*	Yes	34 (27.2)
	Abdominal	20 (16.0)		No	91 (72.8)
	Arthritis	15 (12.0)			
	Headache	14 (11.2)	*Timing of coping*	Reactive	45 (36.0)
	Fibromyalgia	4 (3.2)		Proactive	6 (4.8)
	Multiple	27 (21.6)		Both	5 (4.0)
	^c^Other	3 (2.4)		Not reported	69 (55.2)

*SCD, sickle cell disease; RCT, randomized control trail*.

a *Countries in Europe included The Netherlands (n = 7), Germany (n = 6), UK (n = 5), Sweden (n = 2), Spain (n = 2), Denmark (n = 2), Italy (n = 1), Hungary (n = 1), and Norway (n = 1)*.

b *Other countries included Brazil (n = 2), India (n = 1), Jamaica (n = 1), and Lebanon (n = 1)*.

c *Other pain conditions include complex regional pain syndrome (n = 1;0.8), non-cardiac chest pain (n = 1;0.8), and systemic lupus erythematosus (n = 1;0.8)*.

d *All 32 studies that focused predominantly on an African American sample were concerned with a diagnosis of sickle cell disease*.

e *Other predominant ethnic/racial groups include Hispanic (n = 1), East Indian (n = 1), and Lebanese/Palestinian (n = 1)*.

f *Other designs include non-RCT pre-post design (n = 1), retrospective chart review (n = 1), and ethnography (n = 1)*.

#### Who: Sample Characteristics

The articles included 13,474 youth who were predominately female (*weighted mean* = 65%, *weighted SD* =0.12%). The participants ranged from 3 to 20 years old (*weighted mean* = 12.8 years, *weighted SD* = 2.25).

The participants presented with a wide range of pain conditions (Table 2), with the most frequent being sickle cell disease (SCD; 33.6%) and abdominal pain (16.0%). A heterogenous chronic pain sample was used in 21.6% of studies. Only 78 studies (62.4%) reported on the ethnic and/or racial composition of the participants. When reported, the participants were predominately White (*n* = 41; 54.5%). The only exception was studies on sickle cell disease, wherein the majority of the participants were African American or Black (*n* = 32; 41.0% of studies that reported on ethnicity/race).

#### What: Study Design and Data Collection Method

The majority of included studies examined youth pain-related coping using a cross-sectional (*n* = 85; 67.2%) approach. Of the studies that measured coping as an outcome of a cognitive, behavioral, or physical intervention for youth with chronic pain (*n* = 34), 51.5% (*n* = 17) had a control or comparison group. Additional 16 studies used a control or comparison group without examining the effects of an intervention.

#### Why: Theoretical Frameworks

Just over a third of included studies identified a theoretical conceptualization of coping (*n* = 45; 36.%). Of these 45 studies, the majority (*n* = 29) referred to theoretical frameworks of the coping process (e.g., the biopsychosocial model, risk-resistance model, transactional stress, and coping model) as opposed to conceptualizations of specific coping responses. Five theoretical models conceptualizing specific coping responses were identified within 16 included articles. The most common theory cited was the cognitive-appraisal theory of Lazarus and Folkman (*n* = 8), which suggests that coping responses can be categorized as being “problem-focused” (i.e., coping that is aimed at managing or altering the problem causing the distress) or “emotion-focused” (i.e., coping that is directed at regulating emotional responses to the problem) (Lazarus and Folkman, 1984). Following a similar dichotomous structure, the approach- vs. avoidance framework purposed by Roth and Cohen was used in one study, conceptualizing coping responses as cognitive and emotional strategies that are oriented either toward or away from the stressor, respectively (Roth and Cohen, 1986). Alternatively, five studies referred to the control-based model as a more complex, hierarchical classification system that distinguishes between voluntary vs. involuntary (i.e., unconscious vs. intentional/volitional responses) and engagement vs. disengagement processes (i.e., oriented toward or away from the stressor) (Compas et al., 1991). Within the control-based model, coping is characterized as voluntary responses that can be distinguished by engagement vs. disengagement responses. Engagement responses are further distinguished by their goals: “primary-control-engagement” coping involves attempts to alter emotions (e.g., emotion regulation) or the stressor itself (e.g., problem-solving), whereas “secondary-control-engagement” coping includes efforts to become accustomed to the stressor by modifying cognitions or regulating attention (e.g., acceptance). In contrast, “disengagement” coping involves both removing oneself from the stressor and removing oneself from his or her emotions related to the stressor.

As an alternative to the abovementioned hierarchical classifications of coping responses, one study referred to the typology of Walker and colleagues to explain meaningful patterns of coping responses that are used by youth with chronic abdominal pain and their association with different levels of emotional and physical distress (i.e., coping profiles) (Walker et al., 2008). For example, using cluster analytic techniques with the PRI scales, patients identified as “engaged copers” were characterized by using high levels of “distraction” and “social support-seeking” and associations with lower levels of depressive symptoms and disability, representing an overall adaptive pattern of coping responses. As such, coping profiles were conceptualized to retain information about the specific coping responses while capturing the relationship between coping responses and outcomes.

#### How: Types of Measures

The vast majority of studies employed only quantitative measures (*n* = 108; 86.4%; see Table 2), primarily questionnaires (*n* = 105; see Table 3). Other questionnaires that did not have a peer-reviewed and/or English development/validation study include: the child and adolescent coping inventory (*n* = 1) (Harris et al., 1991); sick-role adoption index (*n* = 1) (Barbarin et al., 1999); Utrecht coping list (*n* = 1) (Westendorp et al., 2017); stress and coping questionnaire for children and adolescents (*n* = 1) and other coping scales derived by the authors of the included study (*n* = 3).

**Table 3:** A list of the questionnaires identified with clear development studies available, reporting psychometrics, ordered by most to least frequently used measure.

**Measure/No. of items**	**Population (P)/Informant (I)**	**Timing**	**Subscales/Factors (Cronbach's alpha, if provided)**	**Response options/Scoring**	**^a^No. of studies**	**^b^Citation accuracy (%)**
Pain Coping Questionnaire (PCQ); 39 items (Reid et al., 1998) Translations: Danish; 36 items (Thastum et al., 1999) Dutch; 39 items (Bandell-Hoekstra et al., 2002) Catalan; 39 items (Huguet et al., 2009) Finnish; 39 items (Marttinen et al., 2018)	P: Healthy youth and youth with recurrent (headache, arthritis) pain, ages 7–17 I: Youth and parent form	Reactive	Approach (0.89) Information seeking (0.79) Problem solving (0.86) Seeking social support (0.86) Positive self-statements (0.82) Problem-focused avoidance (0.86) Behavioral distraction (0.78) Cognitive distraction (0.85) Emotion-focused avoidance (0.85) Externalizing (0.81) Internalizing/Catastrophizing (0.82)	Five-point scale (1 = Never; 5 = Very often)/Factor and subscale scores are derived by computing the means across items or subscales, respectively.	28	96.4
Coping Strategies Questionnaire for SCD (CSQ-SCD); 80 items (Gil et al., 1991) Alternate versions: Original CSQ; 50 items (Rosenstiel and Keefe, 1983)	P: Youth with SCD, ages 7–17 I: Youth and parent form	Reactive	Coping attempts Diverting attention (0.72) Reinterpret pain (0.67) Ignoring pain sensations (0.70) Calming self-statements (0.72) Increased behavioral activity (0.55) Negative thinking Catastrophizing (0.76) Fear self-statements (0.70) Anger self-statements (0.67) Isolation (0.69) Passive adherence Resting (0.72) Taking fluids (0.89) Praying and hoping (0.67) Heat, cold, massage (0.66)	Seven-point scale (Never to Always)/Factor and subscale scores are derived by computing the means across items or subscales, respectively.	21	66.7
Pain Response Inventory (PRI); 60 items (Walker et al., 1997)	P: School-aged sample and youth with recurrent abdominal pain; ages ranged from 8 to 23 I: Youth form	Reactive	Active coping Problem solving Seeking social support Rest Massage/Guard Condition-specific strategies Passive coping Behavior disengagement Self-isolation Catastrophizing Accommodative coping Acceptance Minimizing pain Distract/Ignoring pain Stoicism	Five-point scale (Never to Always)/Factor and subscale scores are derived by computing the means across items or subscales, respectively.	19	100
Pediatric Pain Coping Inventory (PPCI) 41 items (Varni et al., 1996) Alternate version: PPCI–Revised (German) (Hechler et al., 2008)	P: Children and adolescents with musculoskeletal pain, ages 5–16 I: Child, adolescent and parent forms	Reactive	Cognitive self-instruction (0.77) Seek social support (0.74) Strive to rest and be alone (0.73) Cognitive refocusing (0.68) Problem-solving self-efficacy (0.67)	Three-point scale (0 = Not at all, 3 = Often)/Subscale scores are derived by computing the means across items.	7	100
KidCope Adolescent version; 10 items (Spirito et al., 1988) Child version; 15 items (Spirito et al., 1991)	Adolescent: P: High school sample and chronic pain patients, ages 12–18 Child: P: Children, ages 9–13 I: Youth	Reactive	Problem-solving Distraction Social support Social withdrawal Cognitive restructuring Self-criticism Blaming others Emotional regulation Wishful thinking Resignation	Adolescent: Four-point scale (Not at all to Almost all the time) Child: Yes or no Scoring was not specified	6	16.7
Response to Stress Questionnaire (RSQ); 57 items (Connor-Smith et al., 2000)	P: College and high school students and adolescents with recurrent abdominal pain; ages across samples ranged from 12 to 19 I: Youth and parent forms	Reactive	Primary control engagement Problem solving Emotional regulation Emotional expression Secondary control engagement Positive thinking Cognitive restructuring Acceptance Distraction Disengagement coping Denial Avoidance Wishful thinking	Four-point scale (1 = Not at all, 4 = A lot)/Scoring was not specified	5	80
Coping Strategies Inventory (CSI); 72 items (Tobin et al., 1984, 1989)	P: College students I: Self-report	Reactive	Engagement (0.90) Problem-focused engagement (0.87) Problem-solving (0.82) Cognitive restructuring (0.83) Emotion-focused engagement (0.92) Social support (0.89) Express emotions (0.89) Disengagement (0.89) Problem-focused disengagement (0.81) Problem-avoidance (0.72) Wishful thinking (0.78) Emotion-focused disengagement (0.90) Self-criticism (0.81) Social withdrawal (0.94)	Five-point Likert scale (Not at all to Very much)/Factor and subscale scores are derived by computing the sum across items or subscales, respectively.	4	75
The Children's Coping Strategies Checklist (CCSC);45 items (Ayers et al., 1996)	P: School-aged children, ages 8-13 I: Youth	Reactive	Active coping Cognitive decision making Direct problem solving Seeking understanding Positive cognitive restructuring Avoidance coping Cognitive avoidance Avoidant action Distraction Distracting action Physical release of emotion Support Emotion-focused support Problem-focused support	Four-point scale (Never to Most of the time)/Factor and subscale scores are derived by computing the means across items or subscales, respectively.	2	100
Adolescent Coping Style and Behavior (A-COPE); 54 items (Patterson and McCubbin, 1987)	P: Community samples of youth (non-chronic pain), ages 11–18 I: Youth	Reactive	Venting Feelings (0.75) Seeking Diversions (0.75) Developing self-reliance and Optimism (0.69) Developing social support (0.75) Solving family problems (0.75) Avoiding problems (0.71) Seeking spiritual support (0.72) Investing in close friends (0.76) Seeking professional support (0.50) Engaging in demanding activity (0.67) Being humorous (0.72) Relaxing (0.60)	Five-point scale (Never to Most of the time)/Factor and subscale scores are derived by computing the sum across items or subscales, respectively.	1	100
Children's Headache Assessment Scale (CHAS) Versions: Original; 30 items (Budd and Kedesdy, 1989) Revised; 44 items (Budd et al., 1994)	Original: P: Youth with headaches, ages 7-16 Revised: P: Youth with headaches, ages 6-16 I: Parent form	Reactive	Original: Coping response (0.64) Revised: Physical Antecedents and Quiet Coping	Six-point scale (0 = Never, 6 = Always)/Subscale scores are derived by computing the means across items.	1	100
How I Coped Under Pressure Scale (HICUPS); 45 items (Ayers et al., 1996)	P: School sample, ages 9-13 I: Youth	Reactive	Active coping Cognitive decision-making (0.71) Direct problem-solving (0.71) Seeking understanding (0.74) Positive cognitive restructuring (0.62) Avoidance coping Cognitive avoidance (0.61) Avoidant action (0.64) Distraction Distracting action (0.65) Physical release of emotion (0.65) Support Emotion-focused support (0.60) Problem-focused support (0.57)	Four-point scale (Not at all to A lot*)/*Factor and subscale scores are derived by computing the sum across items or subscales, respectively.	1	100
The Schoolagers Coping Strategies Inventory (SCSI); 30 items (Ryan-Wenger, 1990)	P: Community sample (10% with a chronic health condition, e.g., asthma, allergies), ages 8-12 I: Youth	Reactive	Social support Avoidant Emotional Distracting Cognitive Aggressive motor Physical exercise Isolating Aggressive verbal Relaxation Habitual Spiritual Other	Three-point scale (frequency)/Scoring was not specified	1	100
Ways of Coping Checklist; 64 items (Folkman and Lazarus, 1980)	P: Adults, ages 45-64 I: Self-report	Unclear	Problem-focused coping (0.80) Emotion focused coping (0.81)	Yes or No/Scoring was not specified	1	100
Religious Coping (R-COPE); 105 items (Pargament et al., 2000)	P: College sample who encountered a negative life event, ages 18-38; hospitalized adults with a moderately severe medical illness, ages 55-97 I: Self-report	Not reported	Benevolent religious reappraisal (0.91) Punishing God reappraisal (0.92) Demonic reappraisal (0.90) Reappraisal of God's power (0.78) Collaborative religious coping (0.89) Active religious surrender (0.92) Passive religious deferral (0.83) Pleading for direct intercession (0.84) Religious focus (0.84) Purification/forgiveness (0.93) Spiritual connection (0.81) Spiritual discontent (0.88) Marking religious boundaries (0.61) Seeking support from clergy (0.90) Religious helping (0.90) Interpersonal religious discontent (0.82) Religious direction/conversion (0.94)	Four-point scale (Not at all to A great deal*)/*Subscale scores are derived by computing the means across items.	1	100

*The scale characteristics and reliability estimates provided are based on the original scale development study*.

a *A list of each individual study and the study characteristics can be found in Supplementary Table 2*.

b *Citation accuracy refers to the extent to which authors of the included studies cited the correct scale development study*.

Other quantitative measures included structured daily diaries (*n* = 6), structured interviews using closed-end questions (*n* = 2), observations of child behavior (*n* = 1), or an unspecified measure (*n* = 1). Of the quantitative studies, five studies (4.6%) reported using multiple quantitative measures (e.g., a questionnaire and the daily pain and activity diary). Qualitative measures for assessing coping responses included semi-structured interviews with open-ended questions (*n* = 9), unstructured interviews (*n* = 2), drawings (*n* = 1), written narrative tasks (*n* = 1), and clinician judgements based on a retrospective chart review of clinical notes and patient interactions (*n* = 1).

Overall, 75.2% of included studies indicated using child self-report (*n* = 93), and the remaining studies (16.8%) used multiple informants (*n* = 20 child and parent; *n* = 1 child, parent, siblings, and clinicians), parent-reported responses (*n* = 5; 4%), behavioral data (*n* = 1;0.8%), or did not specify (*n* = 5; 4%). Of the 56 studies that explicitly specified the timing of the coping responses assessed in relation to pain, 80.3% indicated measuring reactive coping responses (*n* = 45) and with the remainder reporting on proactive coping (*n* = 6) or both (*n* = 5). Current measures/approaches used to assess proactive coping consisted open-ended interviews (*n* = 5) and daily diary tools (*n* = 6; see Supplementary Table 3).

### Aim 2: Conceptualizations of Coping Responses

A total of 168 coping responses were identified across all included studies (see Supplementary Table 4 for a full list). The following sections summarize: (i) the terminology used for classifying coping responses; (ii) the extent to which coping responses were clearly defined and/or described (conceptual clarity); and (iii) the extent to which authors used a concept-guided approach to measurement selection.

#### Terminology for Classifying Coping Responses

Across the 125 included studies, 21.6% (*n* = 27) reported on only “coping strategies”. Of these studies, the term “coping strategies” was occasionally interchanged with “coping behaviors” or “coping skills”. Furthermore, 38.4% (*n* = 48/125) of the included studies reported on higher-order categories of coping only, of which 75% (*n* = 36/48) did not provide a label for the higher-order categorization. Only seven of these 48 studies (14.5%) explicitly referred to the higher-order categories as “coping styles”. In addition, the term “coping style” was used interchangeably with the following terms: “coping patterns”, “coping approaches”, “dimensions of coping”, “coping subtypes” “coping subthemes”, “domains of coping”, “coping potential”, or “coping response”. In addition, 6.4% (*n* = 8/125) of included studies used a term other than coping strategy or style to categorize coping responses (e.g., coping mechanism, coping behavior, coping skill) and 17.6% (*n* = 22/125) reported on multiple levels of coping responses (e.g., coping strategies and coping styles). Sixteen percent of the included studies (*n* = 20/125) did not provide a specific term to classify the coping responses assessed.

#### Conceptual Clarity

The majority of the 125 studies were rated as “very low” (*n* = 40; 32.0%) or “low” (*n* =19; 15.2%) for conceptual clarity. Forty-seven studies (37.6%) were rated “moderate” because they provided either a definition or specific examples of coping behaviors for each coping response, but not both. As such, it was less common for studies to demonstrate “high” conceptual clarity by including both examples and a specific statement defining the coping responses used (*n* = 19; 15.2%).

#### Measurement Selection

A concept-guided approach to measurement selection was used in only 33.3% of included studies (*n* = 43) as demonstrated by providing a clear and consistent rationale for the chosen measurement tool. Of these 43 studies, 20.9% (*n* = 9) clearly mapped the measurement tool selected to a theoretical framework of coping responses (as described in Aim 2 above). The remaining 79.1% of studies (*n* = 34) were rated as concept guided on the basis that the measure employed clearly mapped onto the study questions, hypotheses, and/or objectives.

### Aim 3: Evaluations of Measurement and Conceptual Consistency (Questionnaires Only)

The majority of studies (76.0%; *n* = 95) assessed coping responses using questionnaires with known psychometric properties (Table 3). Measurement and conceptual consistency were assessed for studies that used questionnaires with known psychometric properties because of their widespread use and the availability of previous scale development or validation studies for comparison. As such, Table 4 presents the list of coping responses, corresponding descriptors, and empirically derived classifications assessed by questionnaires with known psychometric properties.

**Table 4:** A list of coping responses (in alphabetical order) and their corresponding descriptors (if available) conceptualized by questionnaires with a clear scale development study identified (see Table 3 for the list of questionnaires).

**Coping concept**	**Measure**	**Descriptor(s)**	**^a^Factor level**
Acceptance	PRI	No definition was provided. Conceptualized as a coping strategy under “accommodative coping”.	Primary
	RSQ	No definition was provided. Conceptualized as a coping strategy under “secondary control coping”.	
Accommodative coping	PRI	“Strategies, such as positive reappraisal and acceptance, that enable the individual to adapt to unchangeable stressful conditions” (Walker et al., 1997, p. 392). Includes acceptance, self-encouragement, minimizing pain, distract/ignoring pain, and stoicism.	Secondary
Active coping	CCSC/HICUPS	“Strategies in which the child is focused on the stressful event, either to change the situation or to think about it more positively” (Ayers et al., 1996, p. 929). Includes cognitive decision making, direct problem-solving, seeking understanding, and positive cognitive restructuring.	Secondary
	PRI	No definition was provided. Consists of problem-solving, social support, rest, massage/guard, and condition-specific strategies.	
Active religious surrender	RCOPE	“An active giving up of control to God in coping” (Pargament et al., 2000, p. 522)	Primary
Aggressive motor	SCSI	No definition was provided.	Primary
Aggressive verbal relaxation	SCSI	No definition was provided.	Primary
Anger self-statements	CSQ-SCD	No definition was provided. Conceptualized as a pattern of “negative thinking”.	Primary
Approach coping	PCQ	“Direct attempts to deal with the pain and the use of active methods to regulate feelings when in the pain” (Reid et al., 1998, p. 84). Includes information seeking, problem solving, seeking social support, and positive self-statements.	Secondary
Avoidance	CCSC/HICUPS	“Strategies that attempt to manage emotion by trying to avoid or stop thinking about the problem entirely” (Ayers et al., 1996, p. 930). Includes avoidant actions and cognitive avoidance.	Primary (RSQ)/Secondary (CCSC/HICUPS)
	RSQ	No definition was provided. Conceptualized as a coping strategy under “disengagement” coping.	
Avoidant/Avoidant actions/Avoiding problems	A-COPE	“Coping behaviors that involve the use of substances (e.g., drinking beer, smoking) as a way to escape or avoiding persons or issues which cause problems (e.g., staying away from home, telling self the problem is not important)” (Patterson and McCubbin, 1987, p. 174).	Primary
	CCSC/HICUPS	“This includes behavioral efforts to avoid the stressful situation by staying away from it or leaving it” (Ayers et al., 1996, p. 930). Conceptualized as a coping strategy under “avoidance” coping.	
	SCSI	No definition was provided.	
Behavior disengagement	PRI	No definition was provided. Conceptualized as a coping strategy under “passive coping”.	Primary
Behavioral distraction	PCQ	No definition was provided. Conceptualized as a coping strategy under “problem-focused avoidance” coping	Primary
Being humorous	A-COPE	“Coping behaviors focused on not taking the situation too seriously by joking or making “light” of it” (Patterson and McCubbin, 1987, p. 174).	Primary
Benevolent religious reappraisal	RCOPE	“Redefining the stressor through religion as benevolent and potentially beneficial” (Pargament et al., 2000, p. 522).	Primary
Blaming others	KIDCOPE	No definition was provided.	Primary
Calming self-statements	CSQ-SCD	No definition was provided. Conceptualized as a coping strategy under “coping attempts”.	Primary
Cognitive	SCSI	No definition was provided.	Primary
Cognitive avoidance	CCSC/HICUPS	“This includes efforts to avoid thinking about the problem. It includes the use of fantasy or wishful thinking or imagining that the situation was better. It refers to cognitive activity and not behaviors one does to avoid thinking about it” (Ayers et al., 1996, p. 930). Conceptualized as a coping strategy under “avoidance” coping.	Primary
Cognitive decision making	CCSC/HICUPS	“This refers to planning or thinking about ways to solve the problem. It includes thinking about choices, thinking about future consequences, and thinking of ways to solve the problem. It is not simply thinking about the problem but thinking about how to solve it. It involves the planning and not the execution of actions to solve the problem” (Ayers et al., 1996, p. 930). Conceptualized as a coping strategy under “active” coping.	Primary
Cognitive distraction	PCQ	No definition was provided. Conceptualized as a coping strategy under “problem-focused avoidance” coping	Primary
Cognitive refocusing	PPCI	“An active cognitive process to focus one's attention away from pain perception, rather than simply distraction which may imply a more reactive cognitive response to external stimuli” (Varni et al., 1996, p. 148).	Primary
Cognitive restructuring	CSI	“Includes cognitive strategies that alter the meaning of the stressful transaction as it is less threatening, is examined for its positive aspects, is viewed from a new perspective, etc”. (Tobin et al., 1984, p. 2). Conceptualized as a coping strategy under “problem-focused engagement”.	Primary
	KIDCOPE	No definition was provided.	
	RSQ	No definition was provided. Conceptualized as a coping strategy under “secondary control” coping.	
Cognitive self-instruction	PPCI	“Internal self-statements that deal with the child's pain at a cognitive level” (Varni et al., 1996, p. 143).	Primary
Collaborative religious coping	RCOPE	“Seeking control through partnership with God in problem-solving” (Pargament et al., 2000, p. 522).	Primary
Condition-specific strategies	PRI	“Coping strategies specific to abdominal pain, such as going to the bathroom” (Walker et al., 1997, p. 393). Conceptualized as a coping strategy under “active coping”.	Primary
Coping attempts	CSQ-SCD	“Children high on this factor appeared to cope with pain in an active fashion using a variety of cognitive and behavioral coping strategies” (Gil et al., 1991, p. 658). Consists of diverting attention, reinterpret pain, ignoring pain sensations, calming self-statements, and increased behavior activity.	Secondary
Coping response	CHAS	“Thoughts or actions by the child during headaches to help manage them” (Budd and Kedesdy, 1989, p. 3).	Primary
Demonic reappraisal	RCOPE	“The stressor is defined as the work of the devil” (Pargament et al., 2000, p. 522).	Primary
Denial	RSQ	No definition was provided. Conceptualized as a coping strategy under “disengagement” coping.	Primary
Developing self-reliance and optimism	A-COPE	“Coping behaviors focused upon the direct efforts by the adolescent to be more organized and in charge of the situation, as well as to thinking positively about what is happening to him or her (e.g., organizing your life, making your own decisions)” (Patterson and McCubbin, 1987, p. 174).	Primary
Disengagement	CSI	“Strategies that are likely to result in disengaging the individual from the person/environment transaction. Feelings are not shared with others, thoughts about situations are avoided, and behaviors that might change the situation are not initiated” (Tobin et al., 1984, p. 4). Consists of problem avoidance, wishful thinking, social withdrawal, and self-criticism.	Tertiary (CSI/Secondary (RSQ)
	RSQ	“Responses oriented away from a stressor or one's reactions” (Connor-Smith et al., 2000, p. 977). Includes avoidance, denial, and wishful thinking.	
Distraction/Distract and ignoring pain/Distracting/Diverting attention	CCSC/HICUPS	“These strategies are represented by the categories of physical release of emotions and distracting actions. The underlying similarity between these two dimensions of distraction strategies is that the child or adolescent uses some other activity or stimulus to distract themselves from dealing with or thinking about the problem situation” (Ayers et al., 1996, p. 952).	Primary
	CSQ-SCD	No definition was provided. Conceptualized as a coping strategy under “coping attempts”.	
	KIDCOPE	No definition was provided.	
	PRI	No definition was provided. Conceptualized as a coping strategy under “accommodative coping”.	
	RSQ	No definition was provided. Conceptualized as a coping strategy under “secondary control” coping.	
	SCSI	No definition was provided.	
Distracting actions	CCSC/HICUPS	“This includes efforts to avoid thinking about the problem situation by using distracting stimuli, entertainment, or some distracting activity. If the distracting activity involves more than moderate physical exertion it should not be included here” (Ayers et al., 1996, p. 930). Conceptualized as a coping strategy under “distraction” coping.	Primary
Emotion-focused avoidance	PCQ	“Strategies in which emotions are freely expressed and strategies that reflect a lack of effort to regulate feelings when in pain” (Reid et al., 1998, p. 84). Includes internalizing/catastrophizing and externalizing.	Secondary
Emotion-focused coping	Ways of coping checklist	“Cognitive and behavioral efforts directed at reducing or managing emotional distress” (Folkman and Lazarus, 1980, p. 225).	Primary
Emotion-focused disengagement	CSI	“Shutting oneself and one's feelings off from others and criticizing or blaming oneself for what happened” (Tobin et al., 1984, p. 4). Includes social withdrawal and self-criticism.	Secondary
Emotion-focused engagement	CSI	“Items reflect open communication of feelings to others and increased social involvement, especially with family and friends. These coping efforts are focused on the individual's emotional reaction to the stressful situation” (Tobin et al., 1984, p. 3). Includes express emotions and social support.	Secondary
Emotion-focused support	CCSC/HICUPS	“This involves other people in listening to feelings or providing understanding to help the person be less upset” (Ayers et al., 1996, p. 930). Conceptualized as a form of “support seeking”.	Primary
Emotional	SCSI	No definition was provided.	Primary
Emotional regulation	KIDCOPE	No definition was provided.	Primary
	RSQ	No definition was provided. Conceptualized as a coping strategy under “primary control” coping.	
Engagement	CSI	“Attempts by the individual to engage the individual in efforts to manage the stressful person/environment transaction. Through these coping strategies individuals engage in an active and ongoing negotiation with the stressful environment” (Tobin et al., 1984, p. 4). Consists of problem-solving, cognitive restructuring, social support, and express emotions.	Tertiary
Engaging in demanding activity	A-COPE	“Coping behaviors in which poses a challenge from the adolescent to excel at something or achieve a goal such as strenuous physical activity, improving oneself, or working hard on schoolwork” (Patterson and McCubbin, 1987, p. 174).	Primary
Expressing emotions/Express emotions/Emotional expression	CSCC/HICUPS	“This involves the overt expression of feelings either by an action to express feelings, a verbal expression of feelings, or simply an overt release of emotion. It is a solitary activity and does not include discussing feelings with another person. It also does not include inappropriately acting out feelings by threatening or hurting another person” (Ayers et al., 1996, p. 929).	Primary
	CSI	“Releasing and expressing emotions” (Tobin et al., 1984, p. 2). Conceptualized as a coping strategy under “emotion-focused engagement”.	
	RSQ	No definition was provided. Conceptualized as a coping strategy under “primary control” coping.	
Externalizing	PCQ	No definition was provided. Conceptualized as a coping strategy under “emotion-focused avoidance” coping	Primary
Fear self-statements	CSQ-SCD	No definition was provided. Conceptualized as a pattern of “negative thinking”.	Primary
Habitual	SCSI	No definition was provided.	Primary
Heat, cold, massage	CSQ-SCD	No definition was provided. Conceptualized as a coping strategy under “passive adherence”.	Primary
Ignoring pain sensations	CSQ-SCD	No definition was provided. Conceptualized as a coping strategy under “coping attempts”.	Primary
Increased behavioral activity	CSQ-SCD	No definition was provided. Conceptualized as a coping strategy under “coping attempts”.	Primary
Information seeking	PCQ	No definition was provided. Conceptualized as a coping strategy under “approach” coping	Primary
Internalizing/Catastrophizing	CSQ-SCD	No definition was provided. Conceptualized as a pattern of “negative thinking”.	Primary
	PCQ	No definition was provided. Conceptualized as a coping strategy under “emotion-focused avoidance” coping.	
	PRI	No definition was provided. Conceptualized as a coping strategy under “passive coping”.	
Interpersonal religious discontent	RCOPE	“Expressing confusion and dissatisfaction with the relationship of clergy or members to the individual in the stressful situation” (Pargament et al., 2000, p. 524).	Primary
Investing in close friends	A-COPE	“Coping behaviors that involve seeking closeness and understanding from a peer (e.g., be with a boyfriend)” (Patterson and McCubbin, 1987, p. 174).	Primary
Isolating/Isolation	SCSI	No definition was provided.	Primary
	CSQ-SCD	No definition was provided. Conceptualized as a pattern of “negative thinking”.	
Marking religious boundaries	RCOPE	“Clearly demarcating acceptable from unacceptable religious behavior and remaining within religious boundaries” (Pargament et al., 2000, p. 523).	Primary
Massage/Guard	PRI	No definition was provided. Conceptualized as a coping strategy under “active coping”.	Primary
Minimizing pain	PRI	No definition was provided. Conceptualized as a coping strategy under “accommodative coping”.	Primary
Negative thinking	CSQ-SCD	“Children high on this factor appeared to engage in negative thinking patterns including catastrophizing and self-statements of fear and anger, as well as isolation in response to pain” (Gil et al., 1991, p. 658). Consists of catastrophizing, fear self-statements, anger self-statements, and isolation.	Secondary
Passive coping	PRI	No definition was provided. Includes behavior disengagement, self-isolation, and catastrophizing.	Secondary
Passive adherence	CSQ-SCD	“Children high on this factor seemed to rely on concrete coping strategies typically recommended by health care professionals for SCD pain management (e.g., increasing fluid intake, resting), and it appears, they perceive that using these strategies is sufficient for controlling and decreasing their pain” (Gil et al., 1991, p. 658). Consists of resting, taking fluids, praying/hoping, and heat/cold/massage.	Secondary
Passive religious deferral	RCOPE	“Passive waiting for God to control the situation” (Pargament et al., 2000, p. 522).	Primary
Physical exercise	SCSI	No definition was provided.	Primary
Physical release of emotion	CCSC/HICUPS	“This includes efforts to physically work off feelings with physical exercise, play, or efforts to physically relax. There needs to be at least a moderate amount of physical exertion involved, so that very light physical activity for a child (e.g., walking) would not be included here” (Ayers et al., 1996, p. 930). Conceptualized as a coping strategy under “distraction” coping.	Primary
Pleading for direct intercession	RCOPE	“Seeking control indirectly by pleading to God for a miracle or dive intercession” (Pargament et al., 2000, p. 522).	Primary
Positive cognitive restructuring	CCSC/HICUPS	“This refers to thinking about the situation in a more positive way. It includes thoughts that minimize the problem or the consequences of the problem. Acceptance that one can live with the situation the way it is optimistic thinking and an example of positive cognitive restructuring” (Ayers et al., 1996, p. 929). Conceptualized as a coping strategy under “active” coping.	Primary
Positive self-statements	PCQ	No definition was provided. Conceptualized as a coping strategy under “approach” coping.	Primary
Positive thinking	RSQ	No definition was provided. Conceptualized as a coping strategy under “secondary control” coping.	Primary
Praying and hoping	CSQ-SCD	No definition was provided. Conceptualized as a coping strategy under “passive adherence”.	Primary
Primary control engagement	RSQ	“Altering objective conditions such as stressor or one's emotional response to stressor” (Connor-Smith et al., 2000, p. 977). Includes problem solving, emotional regulation, and emotional expression.	Secondary
Problem avoidance	CSI	“The denial of problems and the avoidance of thoughts or action about the stressful event” (Tobin et al., 1984, p. 2). Conceptualized as a coping strategy under “problem-focused disengagement”.	Primary
Problem-focused avoidance	PCQ	“Attempts to disengage from the pain” (Reid et al., 1998, p. 84). Includes cognitive and behavioral distraction.	Secondary
Problem-focused coping	Ways of coping checklist	“Cognitive problem-solving efforts and behavioral strategies for altering or managing the source of the problem” (Folkman and Lazarus, 1980, p. 225).	Primary
Problem-focused disengagement	CSI	“Items reflect denial, avoidance, and an inability or reluctance to look at the situation differently. They reflect cognitive and behavioral strategies to avoid the situation” (Tobin et al., 1984, p. 4). Includes problem avoidance and wishful thinking.	Secondary
Problem-focused engagement	CSI	“Items involve cognitive and behavioral strategies to change the situation or to change the meaning of the situation for the individual. These coping efforts are focused on the stressful situation itself” (Tobin et al., 1984, p. 4). Includes problem-solving and cognitive restructuring.	Secondary
Problem-focused support	CCSC/HICUPS	“Use of other people as resources to assist in seeking solutions, seeking advice/information or direct task assistance” (Ayers et al., 1996, p. 930). Conceptualized as a form of “support seeking”.	Primary
Problem-solving/Direct problem solving	CCSC/HICUPS	“This refers to efforts to change the problem situation by changing the self or by changing the environment. It involves what one does, not what one thinks”(Ayers et al., 1996, p. 929). Conceptualized as a coping strategy under “active” coping	Primary
	CSI	“Behavioral and cognitive strategies designed to eliminate the source of stress by changing the stressful situation” (Tobin et al., 1984, p. 2). Conceptualized as a coping strategy under “problem-focused engagement”.	
	KIDCOPE	No definition was provided.	
	PCQ	No definition was provided. Conceptualized as a coping strategy under “approach” coping	
	PRI	No definition was provided. Conceptualized as a coping strategy under “active coping”.	
	RSQ	No definition was provided. Conceptualized as a coping strategy under “primary control” coping.	
Problem-solving self-efficacy	PPCI	“An adaptive coping strategy consisting of both concrete problem-solving (e.g., put ice or heat on the sore spot) and cognitive statements regarding one's ability to resolve the pain problem (e.g., know that I can do something to main the pain or hurt feel better)” (Varni et al., 1996, p. 149).	Primary
Punishing God reappraisal	RCOPE	“Redefining the stressor as a punishment from God for the individual's sins” (Pargament et al., 2000, p. 522).	Primary
Purification/forgive-ness	RCOPE	“Searching for spiritual cleansing through religious actions” (Pargament et al., 2000, p. 523).	Primary
Reappraisal of God's power	RCOPE	“Redefining God's power to influence the stressful situation” (Pargament et al., 2000, p. 523).	Primary
Reinterpret pain	CSQ-SCD	No definition was provided. Conceptualized as a coping strategy under “coping attempts”.	Primary
Relaxing	A-COPE	“Coping behaviors which focus on ways to reduce tension such as daydreaming, listening to music, or riding around in a car” (Patterson and McCubbin, 1987, p. 174–175).	Primary
Religious direction/conversion	RCOPE	“Looking to religion for assistance in finding a new direction for living when the old one may no longer be viable/looking to religion for a radical change in life” (Pargament et al., 2000, p. 524).	Primary
Religious focus	RCOPE	“Engaging in religious activities to shift focus from the stressor” (Pargament et al., 2000, p. 523).	Primary
Religious helping	RCOPE	“Attempting to provide spiritual support and comfort to others” (Pargament et al., 2000, p. 524).	Primary
Resignation	KIDCOPE	No definition was provided.	Primary
Rest/Resting	CSQ-SCD	No definition was provided. Conceptualized as a coping strategy under “passive adherence”.	Primary
	PRI	No definition was provided. Conceptualized as a coping strategy under “active coping”.	
Secondary control engagement	RSQ	“Focused on adaptation to the problem” (Connor-Smith et al., 2000, p. 977). Includes positive thinking, cognitive restructuring, acceptance and distraction.	Secondary
Seeking diversions	A-COPE	“Coping behaviors focused upon the adolescent's efforts to keep busy and engage in relatively sedate activities that are a way to escape from or forget about the sources of tension and stress such as sleeping, watching TV or reading” (Patterson and McCubbin, 1987, p. 174).	Primary
Seeking professional support	A-COPE	“Coping behaviors directed at getting help and advice from a professional counselor or teacher about difficult problems” (Patterson and McCubbin, 1987, p. 174).	Primary
Seeking social support/Developing social support/Social support	A-COPE	“Coping behaviors directed at efforts to stay emotionally connected with other people through reciprocal problem solving and expression of affect (e.g., helping others solve their problems, taking to a friend about one's feelings, apologizing to others)” (Patterson and McCubbin, 1987, p. 174).	Primary
	CSI	“Includes items that refer to seeking emotional support from people, one's family, and one's friends” (Tobin et al., 1984, p. 2).	
	KIDCOPE	No definition was provided.	
	PCQ	No definition was provided. Conceptualized as a coping strategy under “approach” coping	
	PPCI	“The child seeks aid, comfort, or understanding from parents, peers, and others” (Varni et al., 1996, p. 143).	
	PRI	No definition was provided. Conceptualized as a coping strategy under “active coping”	
	SCSI	No definition was provided.	
Seeking spiritual support/Seeking support from clergy	A-COPE	“Coping behaviors ffocused on religious behaviors (e.g., praying, going to church) or talking to clergy” (Patterson and McCubbin, 1987, p. 174).	Primary
	RCOPE	“Searching for comfort and reassurance through God's love and care” (Pargament et al., 2000, p. 524).	
Seeking understanding	CCSC/HICUPS	“This includes cognitive efforts to find meaning in a stressful situation or to understand it better. It involves seeking understanding of the situation and not seeking to put a positive interpretation on the situation” (Ayers et al., 1996, p. 929). Conceptualized as a coping strategy under “active” coping.	Primary
Self-criticism	CSI	“Blaming oneself for the situation and criticizing oneself” (Tobin et al., 1984, p. 3). Conceptualized as a coping strategy under “emotion-focused disengagement”.	Primary
	KIDCOPE	No definition was provided.	
Self-isolation	PRI	There was no definition provided. Conceptualized as a coping strategy under “passive coping”.	Primary
Social withdrawal	CSI	No definition was provided. Conceptualized as a coping strategy under “emotion-focused disengagement”.	Primary
	KIDCOPE	No definition was provided.	
Solving family problems	A-COPE	“Coping behaviors focused on working out difficult issues with family members (e.g., talk to parent about what bothers you) and doing things with the family” (Patterson and McCubbin, 1987, p. 174).	Primary
Spiritual/Spiritual connection	SCSI	No definition was provided.	Primary
	RCOPE	“Searching for comfort and reassurance through God's love and care” (Pargament et al., 2000, p. 523).	
Spiritual discontent	RCOPE	“Expressing confusion and dissatisfaction with God's relationship to the individual in the stressful situation” (Pargament et al., 2000, p. 523).	Primary
Stoicism	PRI	“Inhibiting or minimizing the expression of one's pain or distress to others” (Walker et al., 1997, p. 393). Conceptualized as a coping strategy under “accommodative coping”.	Primary
Strive to rest and be alone	PPCI	“Attempts to rest or socially withdrawal” (Varni et al., 1996, p. 149).	Primary
Support seeking	CCSC/HICUPS	No definition was provided. Comprised of emotion-focused- and problem-focused support.	Secondary
Taking fluids	CSQ-SCD	No definition was provided. Conceptualized as a coping strategy under “passive adherence”.	Primary
Ventilating feelings	A-COPE	“Coping behaviors focused upon the adolescent's expression of frustrations and tensions such as yelling, blaming others, saying mean things, and complaining to friends or family” (Patterson and McCubbin, 1987, p. 174).	Primary
Wishful thinking	CSI	“Cognitive strategies that reflect an inability or reluctance to reframe or symbolically alter the situation. The items involve hoping and wishing that things could be better” (Tobin et al., 1984, p. 3). Conceptualized as a coping strategy under “problem-focused disengagement”.	Primary
	KIDCOPE	No definition was provided.	
	RSQ	No definition was provided. Conceptualized as a coping strategy under “disengagement” coping.	

*For coping responses captured by multiple questionnaires, descriptors are provided adjacently for ease of comparison. Coping responses are classified as primary, secondary, and tertiary factors based on factor analytic results from the scale development studies. A-COPE, adolescent coping orientation for problem experiences; CSI, coping strategies inventory; CSQ, coping strategies questionnaire; CCSC, children's coping strategies checklist; CHAS, children's headache assessment scale; HICUPS, how I coped under pressure scale; PCQ, pain coping questionnaire; PPCI, pediatric pain coping inventory; PRI, pain response questionnaire; RSQ, response to stress questionnaire; SCSI, schoolagers coping strategies inventory*.

a *An ordered-categorical scale derived from factor analytic results from the scale development studies was used to classify coping responses. The terms “primary”, “secondary”, and “tertiary” correspond to first-, second-, and third-order factor levels, respectively [see Results section “Aim 3: Evaluations of Measurement and Conceptual Consistency (Questionnaires Only), subsection “Classifications”]*.

#### Primary, Secondary, and Tertiary Factor Levels

There were inconsistencies in the terminology used to classify coping responses. For example, higher-order coping responses were referred to by various terms, such as “coping styles”, “coping patterns”, or “coping approaches” (see Section Terminology for Classifying Coping Responses for other examples). Thus, exploratory and/or confirmatory factor analysis results from the original scale development studies used represent the hierarchical classifications of coping responses. The terms “primary”, “secondary”, and “tertiary” correspond to first-, second-, and third-order-factor levels (Table 4). Primary coping responses are made up of related items on a questionnaire. Within multidimensional scales, primary coping responses load onto secondary coping responses; and secondary coping responses load onto tertiary coping responses. For example, the PCQ (Reid et al., 1998) consists of eight primary coping responses (problem-solving, information-seeking, seeking social support, positive self-statements, behavioral distraction, cognitive distraction, externalizing, and internalizing) that each loads onto one of the three secondary factors (approach, problem-focused avoidance, and emotion-focused avoidance); Table 3 presents the factor structure of each questionnaire. In summary, there were 86 primary coping responses (e.g., acceptance, cognitive refocusing), 17 secondary coping responses (e.g., active coping, passive coping), and two tertiary coping responses (i.e., engagement, disengagement). Two coping responses were categorized into multiple levels, depending on the measure used (see Table 4): “avoidance” and “disengagement”.

#### Overall Measurement Consistency

In sum, 55.8% (*n* = 53) of studies were rated “low”, and 23.2% (*n* = 22) were rated “high” for measurement consistency. Measurement consistency was rated as “unclear” for 21.1% of studies (*n* = 20) due to a lack of information about the scale characteristics (i.e., studies that were missing more than two relevant scale characteristics). The most common characteristics missing from articles included the number of items, response options, scoring procedure, and/or the timing of coping strategy use in relation to the pain onset (e.g., proactive vs. reactive).

#### Overall Conceptual Consistency

In terms of conceptual consistency, 35.8% (*n* = 34) of studies were rated “high” (i.e., no discrepancies), and 33.7% (*n* = 32) of studies were rated “low” (i.e., one or more discrepancies). The remaining 30.5% (*n* = 29) of studies were rated as “unclear” because the authors did not provide any descriptors to allow for comparison.

#### Types of Discrepancies

**Figure 3:**
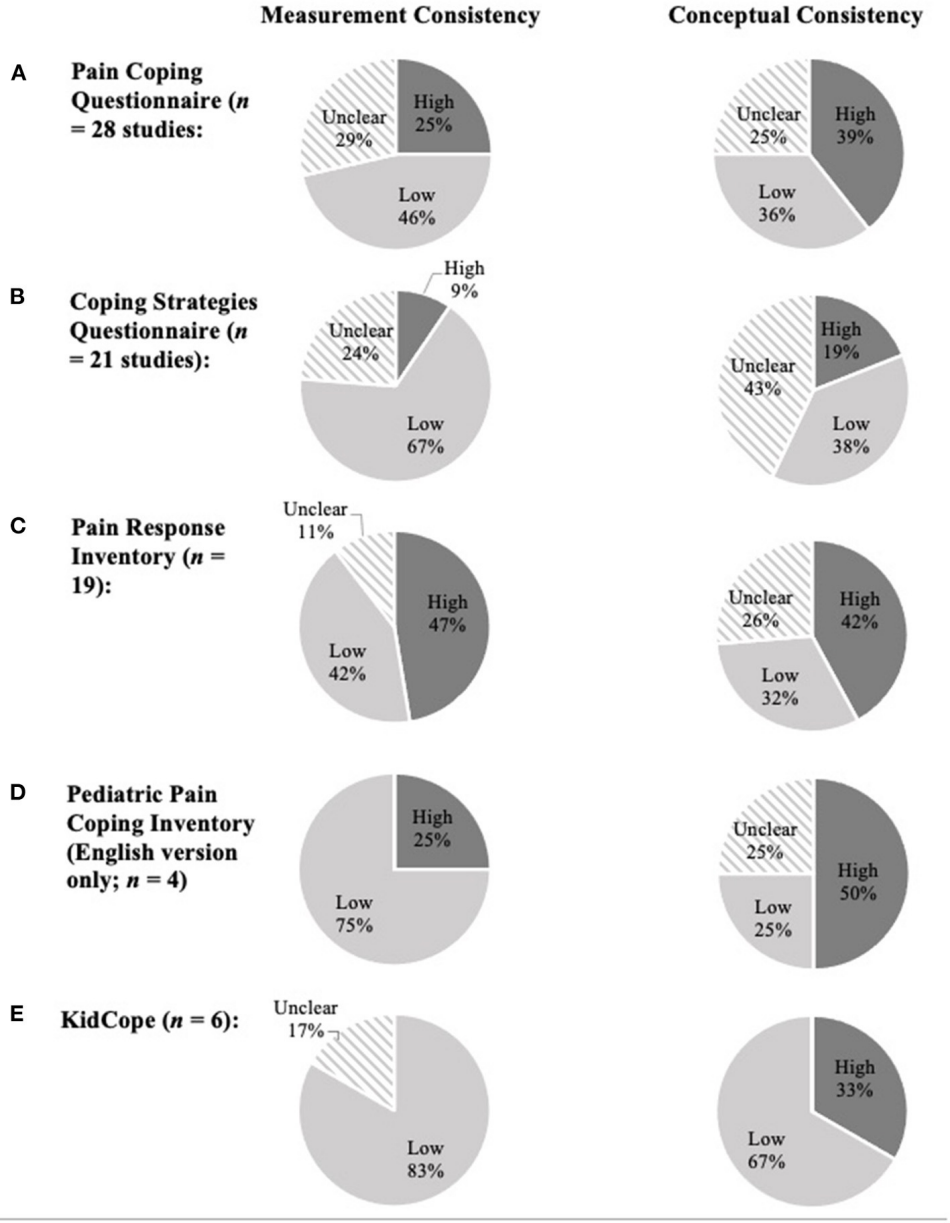
For each measure, overall measurement and conceptual consistency ratings are presented as the percentage (%) of studies that were classified as “high” = no discrepancies, “low” = one or more discrepancies, and “unclear” = unable to evaluate (see full text, Table 1). Charts show the frequency distribution for both consistency ratings for the five most frequently cited questionnaires: **(A)** pain coping questionnaire; **(B)** coping strategies questionnaire; **(C)** pain response inventory; **(D)** pediatric pain-coping inventory (English version); and **(E)** KidCope. “Low” ratings indicate the presence of discrepancies that may impact our ability to directly interpret, consolidate, and/or compare research findings. These ratings do not reflect the quality of the research.

A wide range of discrepancies was identified in the measurement and conceptualization of coping responses by questionnaires across studies. Ratings of measurement and conceptual consistency (Figure 3), and the types of discrepancies (Supplementary Table 5) are summarized for the top five most frequently used questionnaires: PCQ (Reid et al., 1998), CSQ (Gil et al., 1991), PRI (Walker et al., 1997), PPCI (Varni et al., 1996), and KidCope (Spirito et al., 1988, 1991). These five questionnaires were used in 82.7% of studies which employed a questionnaire with known psychometric properties and 64.8% of all included studies. As such, the coping responses assessed by these measures were the focus. Given that the remaining questionnaires were included in few studies, our ability to draw conclusions about their conceptual and measurement consistency is limited and therefore, will not be explored in detail.

##### Pain Coping Questionnaire

Consistent with previous reviews (Blount et al., 2008), the PCQ was the most widely used measure of coping in the context of pediatric chronic pain (ages 7–17; *n* = 28). The PCQ has been used in English (Reid et al., 1998) (24 studies), Dutch (Bandell-Hoekstra et al., 2002) (two studies), and Danish (Thastum et al., 1999) (two studies) samples. Twenty studies provided sufficient information about the PCQ to evaluate measurement consistency, of which 65% (*n* = 13) were found to have at least one discrepancy. Types of discrepancies included using samples outside of the recommended age range of 7–17 years (*n* = 12); alternate composite scores/subscales (*n* = 6); missing one or more subscales (*n* = 2); using different coding for response options (*n* = 2); and using the sum of item-level responses instead of the mean (*n* = 1). Low conceptual consistency was found for 47.6% (*n* = 10) of studies using the PCQ that provided descriptors of the coping responses assessed (*n* = 21). A common discrepancy included the inconsistent categorization of positive self-statements as a type of “problem-focused avoidance” instead of “approach” coping (*n* = 3). In addition, several studies used different labels for coping constructs, such as “distraction” instead of “problem-focused avoidance” (*n* = 4) and “positive approach” in place of “approach coping” (*n* = 1).

##### Coping Strategies Questionnaire for Sickle Cell Disease

Of the studies that provided sufficient information about the measurement characteristics of the coping strategies questionnaire for sickle cell disease (CSQ-SCD) (*n* = 16), 87.5% (*n* = 14) had at least one discrepancy. The most common discrepancy was the inclusion of participants beyond the age range of the scale development study (i.e., 7–17 years old; *n* = 13). Other discrepancies included using participants with a pain condition other than SCD (*n* = 2); alternate composite scores (e.g., total coping attempts, rational thinking; *n* = 3); missing subscales (*n* = 1); using a six- instead of seven-point Likert scale (*n* = 2); using a 44-item version of the CSQ-SCD (*n* = 2); and/or computing subscales by taking the sum of the item-level responses instead of the mean (*n* = 2). Conceptual consistency was evaluated for 12 of the included studies that used the CSQ-SCD. Of these studies, 66.7% (*n* = 8) reported a discrepancy in their conceptualization of particular coping responses. The primary reason for low ratings of conceptual consistency was the use of alternate terminology for constructs operationalized by the CSQ-SCD (*n* = 4). For example, the terms “illness-focused strategy” and “adherence” have been used in place of “passive adherence”.

##### Pain Response Inventory

Upon evaluating measurement consistency (*n* = 17), 47.1% (*n* = 8) of studies had more than one discrepancy. The reasons for low ratings of measurement consistency included the following: the inclusion of participants outside of the age range of the PRI validation sample (i.e., 7–17 years old; *n* = 3); used in a sample of youth with pain conditions other than abdominal pain (*n* = 2); computing subscales by taking the sum of the item-level responses instead of the mean *(n* = 1); missing subscales (*n* = 4); and/or alternate categorizations of subscales (*n* = 1). Conceptual consistency was evaluated for 14 studies using the PRI. The original scale development study of the PRI did not provide definitions of the coping constructs assessed, and therefore, conceptual consistency was evaluated by the inclusion of specific examples of coping strategies that fall within each of the higher-order factors. As such, a common discrepancy was the exclusion or incorrect categorization of lower-order subscales in the conceptualization of higher-order ones (*n* = 5). For example, several studies excluded “stoicism” in the conceptualization of “accommodative coping” (*n* = 3). In addition, inconsistent labeling of coping responses was demonstrated by one study that used the term “activity restriction” in place of “behavior disengagement”.

##### Pediatric Pain Coping Inventory

Measurement and conceptual consistency could not be evaluated for studies that used the German version of the Pediatric Pain Coping Inventory (PPCI) because the scale development study was not available in English. As a result, four studies were used to evaluate measurement and conceptual consistency for the PPCI. In terms of measurement consistency, 75% of studies that used the PPCI (*n* = 3) were rated “low”. Types of discrepancies included the inclusion of participants beyond the intended age range (i.e., 5–16 years old; *n* = 2) and pain type (*n* = 1) validated for the PPCI. Moreover, conceptual consistency was rated “low” for one study (25%) that used the PPCI. This study reported using the *a priori* coping responses (“distraction”, “problem-solving”, and “helplessness”) as opposed to those supported by factor analysis and psychometric properties of the PPCI assessed in children with chronic pain (“strive for rest and be alone”, “cognitive refocusing”, and “problem-solving self-efficacy”).

##### KidCope

The majority of the six studies (83.3%) that employed the KidCope were rated low for measurement consistency. The most common discrepancy was the inclusion of participants aged two or more years outside of the age range validated for the KidCope (i.e., child version = 9–13; adolescent version = 12–18) (*n* = 5). While the KidCope was developed as a unidimensional scale, two studies computed alternate higher-order scores using the coping strategies measured by the KidCope. In addition, one study used a four-point Likert scale for the adolescent version rather than the original “yes/no” response items. With regard to conceptual consistency, 66.7% of studies that used the KidCope were rated as “low”. Two studies proposed using the coping responses assessed by the KidCope to conceptualize “active” and “passive” coping; and, in contrast, one other study conceptualized “negative/avoidance” and “positive/approach”. Neither of these higher-order conceptualizations of coping responses was proposed in the development of the KidCope. In another study, discrepancies included mislabeling coping responses (e.g., using the term “withdrawal” in place of “social withdrawal”) or adding coping responses that are not typically conceptualized by the KidCope (i.e., remain positive, blame self instead of self-criticism, express emotions, relax, become helpless, different feelings, focus on future, taking medication/laying down).

## Discussion

This scoping review is the first to examine the sample and methodological characteristics, theoretical frameworks, and measurement tools used to measure and conceptualize coping in pediatric chronic pain. Overall, a lack of theory, conceptual clarity, conceptual consistency, and measurement consistency emerged in included studies. In the discussion, these results and their implications are explored in relation to four major gaps and are drawn upon to offer recommendations for future research.

### Gap 1: Lack of Theory, Conceptual Clarity, and Conceptual Consistency

It is important for researchers to ground their research questions and methods in theory to ensure an appropriate use of measures and concepts. Also, explicit use of theory helps readers to make sense of and interpret findings. Less than 13% of included studies provided a clear explanation of a theoretical framework for the coping responses assessed. Even fewer studies (7%) used theory to justify the research methods employed. Current theoretical frameworks focus on the conceptualization of higher-order coping responses (i.e., secondary factors such as emotion-focused vs. problem-focused coping, tertiary factors such as engagement vs. disengagement) and organize lower-order coping responses within them (i.e., primary factors). The overreliance on quantitative and exploratory methods has contributed to an excessive number of lower-order conceptualizations (86 primary coping responses) that makes it challenging to synthesize, interpret, and apply this literature in future research and clinical contexts. Thus, theoretical frameworks for primary coping responses are needed to clearly and consistently operationalize overt thoughts and behaviors used for coping. Building and incorporating strong theoretical frameworks could help to establish a more parsimonious literature by avoiding redundant and synonymous conceptualizations (e.g., “ventilating feelings” and “expression of emotion”).

The use of the terms “coping styles” to refer to higher-order categories was not as prominent as expected, and 75% of studies that focused on conceptualizing higher-order categories did not use any particular coping terminology to describe them. As such, a more explicit use of theory can also bring clarity and consistency to the conceptualization of higher- vs. lower-order categories of coping responses. For example, some researchers might use *dispositional/stylistic* categories, such as “coping styles”, if the recommendation is to view coping responses as being stable over time and situation (Moos and Holahan, 2003). In contrast, researchers might avoid specific or highly restrictive terminology when using a *contextual* perspective, wherein coping responses are used differently in response to developmental (e.g., maturity) and environmental factors (e.g., access to resources) (Roberts et al., 2001; Moos and Holahan, 2003; Kim-Cohen et al., 2004). An *integrative* perspective might view some coping responses as stable and others as flexible over time and situations. As a field, it is important to further explore whether a particular theory or an integrative approach could drive a more clear and consistent understanding of the terminology used to refer to hierarchical classifications of coping responses.

Almost half of the included studies did not explicitly state descriptors of the coping responses assessed. Also, a third of the studies that used questionnaires described coping responses in a way that was inconsistent with the original scale development study, such as using a different definition(s), categorizations of lower-order into higher-order coping responses, or alternate terminologies for referring to factors. One issue contributing to low conceptual clarity and consistency is the tendency for authors to describe coping responses based on their relationship with coping *outcomes* rather than the *nature* or operationalization of the thoughts or behaviors underlying the coping response. For example, some authors have described both “passive coping” and “passive adherence” solely by their associations with maladaptive outcomes in youth with chronic pain (e.g., Thompson et al., 1994; Logan et al., 2012). This description is problematic for two main reasons. First, coping responses have inconsistent relationships with coping outcomes, and the factors contributing to these inconsistencies are not well-understood (Skinner et al., 2003; Zimmer-Gembeck and Skinner, 2016). Therefore, while “passive coping” and “passive adherence” are generally associated with negative outcomes, this might not be consistent across studies, individuals, or contexts. Second, relying on this description alone does not provide any information about what it looks like for a youth to use either of these coping responses and can lead to confusion between these two terms that are conceptually distinct (for an example, see Figure 1). While the adaptive and maladaptive qualities of coping responses are relevant to their conceptualization, researchers should prioritize using definitions and examples in their papers that help readers to reproduce these concepts in research and discuss them in clinical practice.

### Gap 2: Lack of Diverse Research Methods and Measurement Consistency

Most of our knowledge about coping in the context of pediatric chronic pain is based on quantitative methods (85%) and cross-sectional designs (67%) with an emphasis on parent- and/or self-report questionnaires to assess coping responses in youth (86%). The five most common questionnaires identified were four pain-specific coping questionnaires (i.e., PCQ, PRI, PPCI, and CSQ-SCD) and one general coping questionnaire (i.e., KidCope). The use of questionnaires to assess coping is, generally, a cost-effective and convenient method for data collection. Some questionnaires (e.g., PCQ, KidCope) are available in different versions (e.g., language, age, and pain condition), enabling more tailored selection. Moreover, questionnaires can be used to promote more consistent conceptualizations of coping in the literature and allow for the consolidation of research findings across studies if used frequently and consistently by study authors. Unfortunately, regarding measurement consistency, just over half of studies had one or more discrepancies, and one in five was missing more than two relevant scale characteristics. However, as we employed a strict criterion for measurement consistency, a “discrepancy” does not necessarily mean that the study was flawed or inaccurate but rather highlights an inconsistency between studies that may impact our ability to directly consolidate and/or compare research findings. In addition, the high proportion of studies missing information about the scale in their methods highlights a lack of transparency in the literature, which in turn, limits the reproducibility of studies and the ability to confidently consolidate findings across studies. The less we can consolidate findings, the less we can advance the field both in terms of theory building and testing as well as interventions.

Another potential limitation of questionnaires is the possibility for psychometric inadequacies, including an unstable or unsubstantiated factor structure (e.g., over-factoring, poor reproducibility), inadequate or non-existent construct validity, and no reports of test-retest reliability (Parker and Endler, 1992; Blount et al., 2008). Additionally, the use of exploratory factor analytic procedures contributes to an abundance of complex and difficult-to-interpret constructs that lack clinical utility and relevance (Parker and Endler, 1992; Blount et al., 2008). While overarching categories of coping responses (i.e., factors) can be useful for understanding and predicting coping outcomes, these constructs can be abstract and difficult to operationalize in the development of interventions (Blount et al., 2008). As such, Blount and colleagues (Blount et al., 2008) highlighted the importance of assessing and reporting on discrete, trainable mental actions or behaviors. One approach to assessing specific coping behaviors is by analyzing and interpreting item-level responses on questionnaires (Blount et al., 2008; Schwartz, 2016). For example, using the PCQ, researchers and clinicians might examine group or individual differences in responses to “talk to a family member about how I feel” and “talk to a friend about how I feel” instead of the subscale score for “seeking social support”. This information helps to understand the specific ways that social support is used by youth to cope with pain. Although item-level responses are less psychometrically sound and provide less information about the latent variable being measured, they can be relevant to intervention studies where outcomes are used to inform intervention training and clinical recommendations. Alternatively, behavioral assessment tools are useful for assessing discrete, overt coping behavior (e.g., observable body movements, sounds, and words), which can be advantageous for identifying and monitoring specific behaviors, overcoming barriers to self-report (e.g., social desirability), and working with youth who have complex developmental or intellectual disabilities. To date, in contrast to acute pain, there are no well-established behavioral coping measures for pediatric *chronic* pain (Blount et al., 2008; Chorney and McMurtry, 2014), and therefore, developing such tools is an important direction for future research.

### Gap 3: Poor Understanding of Coping Responses in Diverse Patient Populations

Although participant characteristics varied across studies in terms of age (3–20 years) and pain conditions, the lack of research in specific patient populations limits the validity of current measurement tools and conceptualizations of coping responses in certain populations. There is evidence to suggest that the use of coping responses may be influenced by age (Curry and Russ, 1985; Compas, 1998; Dubow and Rubinlicht, 2011). For example, young children may rely more on parental support for coping, whereas adolescents have a greater capacity for using more cognitive-oriented coping responses (e.g., cognitive restructuring) and for seeking a broader array of informational, emotional, and tangible supports beyond the family (Dubow and Rubinlicht, 2011). However, very few studies focused exclusively on children under 12 years with chronic pain (8%), and most studies included both child and adolescent participants (68%). Of note, no studies on coping in infants and toddlers with chronic pain were identified nor measures with known psychometric properties for children under 5 years. It is unclear whether this lack of research stems from actual low prevalence of chronic pain in infants (estimated at 1–3%) (Perquin et al., 2000; King et al., 2011) or challenges with detecting and adequately describing chronic pain in infancy (Pillai Riddell et al., 2009; DiLorenzo et al., 2016). Also, current models of conceptualizing coping responses as voluntary/intentional thoughts or actions may be inappropriate to understand infant coping as they have limited cognitive and language capacity as well as learned experiences to independently implement coping responses or describe their ways of coping. More nuanced research is needed to better understand how coping responses may change across development.

Studies were primarily conducted in the United States or Europe (88%) and included predominately female participants (65%). Apart from studies focused on youth with sickle cell disease [which primarily affects individuals of African or Caribbean descents (Hassell, 2010)], the majority of studies that reported on race/ethnicity included white participants (93%). This highlights a lack of measurement and conceptualization of coping responses in diverse cultural (i.e., cross-national, cross-ethnic, and cross-racial cultures) and/or sex/gender groups of youth with chronic pain. A systematic review of the adult chronic pain literature suggests that mean scores on measures of pain-related coping responses vary significantly between people of different countries and languages (Sharma et al., 2020). In addition, cultural and contextual theories of coping highlight that while stress and coping, in general, are universal experiences, members of different cultures might not only experience different/additional stressors but also consider and respond to stressors differently with respect to coping goals, responses, and outcomes (Kuo, 2011). For example, members of individualistic cultures tend to prioritize “externally targeted control” (i.e., changing the environment/stressor), whereas members of collectivistic culture tend to use “internally targeted control” (i.e., changing oneself) (Kuo, 2011). This highlights the importance of understanding which coping responses are relevant to assess and recommend for diverse youth.

Although there is evidence for sex-specific engagement in coping responses among adolescents with chronic pain (e.g., females reported greater use of social support networks than males) (Keogh and Eccleston, 2006), this research has not explored the biological and sociocultural mechanisms for these group differences. A poor understanding of the underlying mechanisms of sex differences and the interchangeable use of the terms “gender” and “sex” in this research poses risks for sex-based stereotypes regarding the effectiveness of certain coping responses that may not be consistent with individual preferences and/or gender identity (Boerner et al., 2018; Samulowitz et al., 2018). Important gaps to be addressed in the literature include considering the role of gender identity/expression in coping as well as to make clear in research papers when birth-assigned sex vs. self-identified gender is being reported.

### Gap 4: Lack of Measurement Tools and Conceptualizations for Proactive Coping

Few studies (<6%) assessed proactive coping responses using either interview or daily diary-recorded measures. Although research on proactive coping is limited in youth (Schwarzer and Luszczynska, 2008), studies in adults demonstrate that proactive coping is associated with various positive psychological (e.g., higher life satisfaction) and physical outcomes (e.g., rehabilitative functioning) (Katter and Greenglass, 2013; Miao et al., 2017; Bhattacharyya et al., 2018). Moreover, the concept of proactive coping is consistent with the push for long-term behavioral changes in the management of chronic pain (Landry et al., 2015; Miró et al., 2017). Taken together, proactive coping is deemed a promising approach to coping for youth with chronic pain.

To advance our ability to assess proactive coping, it is important to first clarify how to conceptualize proactive coping responses *via* theory. Thus, more qualitative research in this area would be useful for identifying different proactive coping responses. Based on this information, the next step would be to derive appropriate assessment tools that capture these responses. To date, the proactive coping inventory (PCI) (Greenglass et al., 1999) for adults is the only known questionnaire for assessing proactive coping responses. Perhaps, the adult-focused literature on proactive coping stems from the need for individuals to be able to anticipate pain-related stressors and independently implement lifestyle changes. As such, self-report assessments may be useful for older youth who may have accumulated experience with chronic pain and can take an active role in implementing lifestyle changes. In contrast, researchers may consider the role of parents in assessments for young children. A greater capacity for understanding and assessing proactive coping in the youth of all ages holds promise for preventing the impact of chronic pain on overall health and psychosocial well-being.

### Recommendations for Future Research

**Figure 4:**
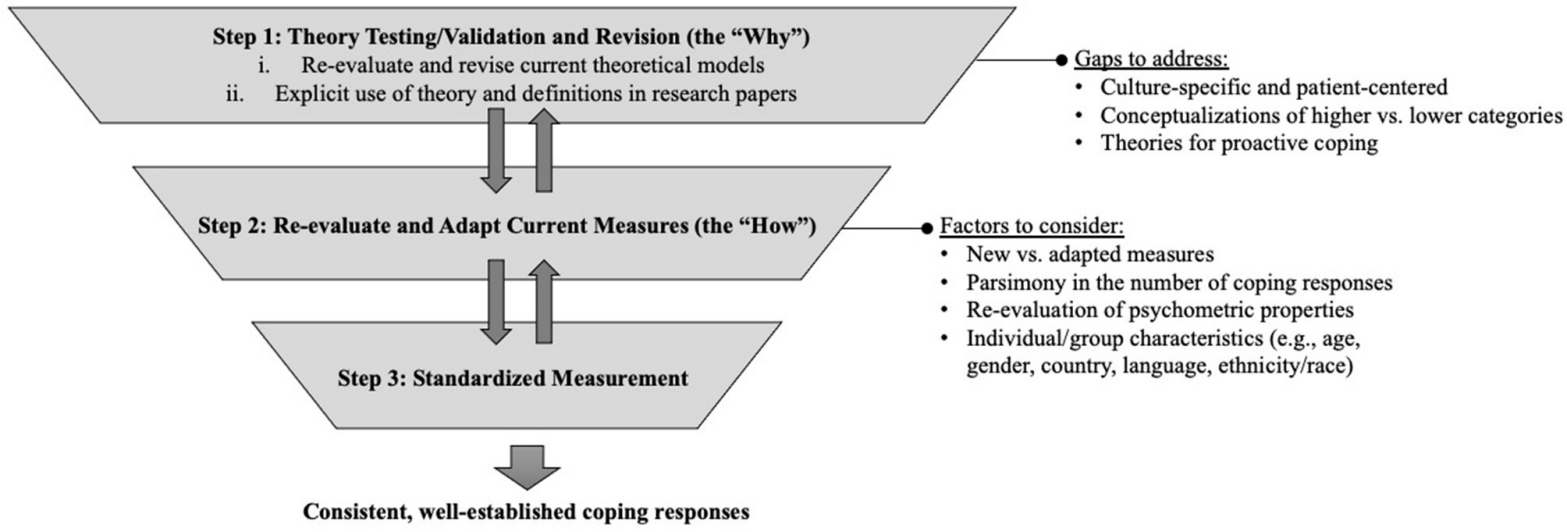
Proposed three steps for future research aimed at establishing more consistent, well-defined coping responses. Double arrows indicate the iterative and interactional relationship between steps.

The following sections of this review outline a proposed three-step process (Figure 4) for establishing more clarity and consistency in the literature.

#### Step One: Theory Testing/Validation and Revision

There is a critical need for more explicit, well-developed theories of coping in the pediatric chronic pain literature that can then provide the knowledge base for construct validation (Strauss and Smith, 2009). Theoretical frameworks serve as a rationale for *why* we measure particular coping responses by clarifying their meaning and conceptual relationships. Theories need to be culture specific and patient centered (Kuo, 2011). In addition, advancements in theory should clarify how we think about and relate higher- and lower-order coping responses (e.g., dispositional vs. conceptual frameworks).

Current theories of coping used in pediatric chronic pain research were derived from adult or non-chronic pain populations and were rarely cited in the research literature. As such, two pertinent steps need to be taken. First, considering that theory testing/validation is an iterative process (Strauss and Smith, 2009), there is a need for research aimed at either re-evaluating and revising current theoretical models of pain-related coping responses to be applicable to pediatric chronic pain or beginning anew. In these efforts, ways of conceptualizing proactive coping responses should be explored. Assimilating qualitative and/or observational methodologies may prove particularly helpful to test theoretical mechanisms and better capture the lived experiences of youth with chronic pain (Tutelman and Webster, 2020).

Second, it is important to recognize that theories and data are independent of each other (Strauss and Smith, 2009); therefore, researchers are responsible for drawing connections between their findings and theory to improve the clarity and consistency of literature. This would include using more explicit statements of theory and definitions in the introduction or the discussion section of a paper that would: (i) clarify the relevance of particular coping responses to the population of interest; (ii) justify the use of a given measure; (iii) promote a mutual understanding of research findings; and (iv) inform future efforts toward theory validation (Parker, 2020). The underuse or superficial use of theories and definitions in papers may relate, in part, to manuscript length restrictions put in place by publishers. In these situations, authors should consider the use of supplementary materials or open access repositories to share additional conceptual information needed.

#### Step Two: Measure Development, Evaluation, and Modification

Clear and well-defined theories should be used to guide *how* we measure coping responses. Although the development of new theories may dictate the need for new measures, researchers should, first, carefully consider whether existing measures can be adapted to fit new/modified theories. Adapting existing measures would be beneficial for reassessing the validity of former coping responses, avoiding the creation of redundant or interchangeable coping responses, and comparing new and extant research. More parsimonious literature can also be established by cross-validating or performing content analyses between different coping measures (Crombez et al., 2020). Measures of coping should be tested and periodically re-evaluated in samples of youth with chronic pain, which, in turn, may serve to validate and/or revise the theoretical frameworks used. As such, the process of developing/modifying and evaluating measures (Step 2) should be iterative and interactive with theory construction (Step 1).

This review highlights the need for research aimed at evaluating the reliability, validity, and clinical utility of measures and conceptualizations of coping responses in different age, gender, and cultural groups. In other fields, the use of item content analyses, focus groups, or cognitive interviews has been useful for capturing group differences and patient experiences using particular measures (Beatty and Willis, 2007; Amtmann et al., 2018; Crombez et al., 2020). Researchers have also used culture-specific and patient-centered approaches for recruitment to increase the diversity of youth in research (Zamora et al., 2016; Winter et al., 2018). Alternatively, the recruitment of sufficient sample sizes can allow for age, gender, and ethnicity-specific analyses (Winter et al., 2018).

#### Step Three: Standardized Measurement

Once the field develops fewer, more well-established measures with clearly defined coping responses grounded in theory, researchers may consider the benefits of using a publicly available registry. This uniform approach has been adopted by the patient-reported outcomes measurement information system (PROMIS^®^) to measure pain sensations (i.e., intensity and quality), interference, and behaviors in the context of chronic pain (Jacobson et al., 2015; Askew et al., 2016; Witter, 2016; Singh et al., 2019). Registered coping measures specific to youth with chronic pain may help to establish a unified understanding of coping, meaningful and relevant measurement tools, and comparability across studies.

### Recommendations for Clinical Practice

Although considerable work is needed to improve the clarity and clinical utility of the pediatric pain-coping literature, this review can serve as a resource for clinicians. For instance, clinicians can use this review to: (1) access a list of specific coping responses; (2) locate relevant research; and (3) identify and select measures for assessing the use of pain-coping responses in youth. However, there is currently, no best way to measure and conceptualize coping responses in this population. Instead, clinicians are encouraged to select measurement tools that have strong psychometric properties when used with their target population (i.e., age, pain condition) and based on their content (e.g., specific questions). For example, the PRI was developed for use in youth with abdominal pain, and therefore, a clinician working in a gastroenterology service may implement the PRI in his or her clinical practice. Alternatively, by comparing the item-level content for “seeking social support” on the PRI and PCQ, for example, it is apparent that the PCQ is better able to distinguish between the types of companions (friends vs. family) than the PRI. Therefore, the clinician may select the measure based on the goals of his or her assessment. Regardless, clinicians are encouraged to be intentional about their measure choice. In addition, clinicians can supplement their assessments with open-ended questions that create opportunities for patients to share information about more culturally relevant and person-specific approaches to coping. Furthermore, clinicians can play an important role in building consistency to the field by using evidence-based terminologies and conceptualizations of coping responses with their patients in a way that aligns with how they are intended to be used.

## Strengths and Limitations

This review is the first to evaluate the clarity and consistency of the measurement and conceptualization of coping responses in youth with chronic pain. Strengths include the comprehensive search strategy and rigorous methodology used to identify and summarize published research. The findings of this review will allow researchers to design studies to address research gaps and inform more consistent and targeted assessments and conceptualizations of coping responses.

There are also limitations. One inherent limitation of a scoping review approach is that it does not formally evaluate the quality of evidence or allow for conducting a comprehensive synthesis of research findings (Pham et al., 2014; Munn et al., 2018). Therefore, concrete guidance on how best to measure or reconceptualize coping responses in the literature is beyond the scope of this review. In addition, this review did not consider the order and mode of administration (e.g., online, paper, and verbal), which may impact the types of coping responses reported by youth (Bowling, 2005). Also, we did not include a comparison between measures; however, it is critical to consider how assessments of frequency vs. duration vs. intensity may influence how youth report on the same coping response (Stone et al., 1991).

Another limitation is the potential magnification of the extent of discrepancies. A set of strict criteria was used due to the lack of clarity as to which discrepancies hinder the reliability and validity of the results. For example, the most common discrepancy for measurement consistency was using a sample outside the age range validated by the scale. The extent to which a particular measure can be used in a sample of participants even 1 year outside of the age range is unclear.

Furthermore, there are factors that may have limited the breadth of this review. For instance, this review did not take into account studies that were not available in English, which may have limited the inclusion of research on geographically and ethnically diverse patient populations. In addition, coping is a nebulous concept; the distinction between thoughts and behaviors that would constitute coping as opposed to adaptation or self-management is not always obvious (Audulv et al., 2016). Likewise, coping responses are often confused with interventions for chronic pain (e.g., mindfulness training, medications, and exercise therapy) or self-care routines (e.g., getting enough sleep). Given that this review attempted to focus exclusively on studies that measured coping responses, the lack of clarity of how to differentiate coping responses from similar concepts may have led to the exclusion of relevant studies.

## Conclusion

This review was a necessary first step toward providing concrete guidance on how best to measure or reconceptualize coping responses in the literature on pediatric chronic pain. The results demonstrate the complexity of the literature and highlight gaps and inconsistencies across studies. These gaps are underscored by the lack of theories and definitions/examples of coping responses across studies, which makes it challenging to interpret and apply research findings. Additionally, the wide range of measurement tools and inconsistencies in their use further contributes to the confusing state of this literature. It is recommended that future research prioritize the development and testing of theories and measures of coping responses for pediatric chronic pain. These efforts should be an iterative and interactive process and include a wider range of participants and cultures. Ultimately, the implementation of standardized measures grounded in theory with clear definitions of coping responses is critical to establish clearer and more consistent conceptualizations of coping in the field.

## Data Availability Statement

The original contributions presented in the study are included in the article/Supplementary Material, further inquiries can be directed to the corresponding author/s.

## Author Contributions

This work was conducted as the master's thesis of ANN under the supervision of CMM. BAM is an advisory committee member. ANN, RMT, and CMM conceived the project with feedback from BAM. ANN and RMT conducted the searches. ANN analyzed results and wrote the manuscript. All authors read, edited, and approved the final manuscript.

## Funding

This work was supported by the general purpose research account of CMM from the University of Guelph and infrastructure funded by the Canadian Foundation for Innovation and the Ministry for Research and Innovation. ANN was supported by the Ontario Graduate Scholarship (from 2019 to 2021).

## Conflict of Interest

The authors declare that the research was conducted in the absence of any commercial or financial relationships that could be construed as a potential conflict of interest.

## Publisher's Note

All claims expressed in this article are solely those of the authors and do not necessarily represent those of their affiliated organizations, or those of the publisher, the editors and the reviewers. Any product that may be evaluated in this article, or claim that may be made by its manufacturer, is not guaranteed or endorsed by the publisher.
