# Supplementary material for: The Measurement and Conceptualization of Coping Responses in Pediatric Chronic Pain Populations: A Scoping Review
Source: Front Psychol. 2021 Oct 1;12:680277. doi: 10.3389/fpsyg.2021.680277 (PMC8519346; doi:10.3389/fpsyg.2021.680277)
Supplement: Supplementary file 1 [file Data_Sheet_1.docx]

Supplementary Material

# Search Strategy and Syntax

The following search strategy was taken from larger review on positive psychological factors in the context of pediatric chronic pain (see citation below). A list of search terms was developed through an iterative process of consulting the extant literature, expert collaborators, and a social sciences librarian. The resulting search strategy and syntax was used in two electronic bibliographic databases to identify articles on coping with pediatric chronic pain published up until August 20^th^, 2020.

Citation: Tomlinson R. *Positive Psychological Factors in Pediatric Chronic Pain* (Doctoral dissertation). http://hdl.handle.net/10214/16939

**PsycINFO**

1. ti=pediatric* or it=pediatric* or ab=pediatric*

2. ti=paediatric* or ab=paediatric*

3. ti=adolesc* or it=adolesc* or ab=adolesc*

4. ti=child* or it=child* or ab=child*

5. ti=infan* or it=infan* or ab=infan*

6. ti=youth or it=youth or ab=youth

7. 1 or 2 or 3 or 4 or 5 or 6

8. ti=pain or it=pain or ab=pain

9. ti=headache or it=headache or ab=headache

10. ti=migraine or it=migraine or ab=migraine

11. ti=“stomach ache” or ab=“stomach ache”

12. ti=stomachache or ab=stomachache

13. ti=“tummy ache”

14. ab=tummyache

15. ab=bellyache

16. ab=earache

17. ab=“ear ache”

18. ab=“tooth ache”

19. ti=toothache or ab=toothache

20. ti=odontalgi* or ab=odontalgi*

21. ti=dysmenorr* or it=dysmenorr* or ab=dysmenorr*

22. ti=neuralgi* or it=neuralgi* or ab=neuralgi*

23. ti=fibromyalgia or it=fibromyalgia or ab=fibromyalgia

24. ti=arthritis or it=arthritis or ab=arthritis

25. ti=“sickle cell” or it=“sickle cell” or ab=“sickle cell”

26. ab=sicklecell

27. 8 or 9 or 10 or 11 or 12 or 13 or 14 or 15 or 16 or 17 or 18 or 19 or 20 or 21 or 22 or 23 or 24 or 25 or 26

28. ti=“quantitative sensory testing” or ab=“quantitative sensory testing”

29. ti=needle* or it=needle* or ab=needle*

30. ti=“cold pressor” or ab=“cold pressor”

31. 28 or 29 or 30

32. ti=adapt* or it=adapt* or ab=adapt*

33. ti=resilien* or it=resilien* or ab=resilien*

34. ti=wellbeing or ab=wellbeing

35. ti=well-being or it=well-being or ab=well-being

36. ti=optimis* or it=optimis* or ab=optimis*

37. ti=hope or it=hope or ab=hope

38. ti=acceptance or it=acceptance or ab=acceptance

39. ti=“psychological flexibility” or ab=“psychological flexibility”

40. ti=“positive affect” or ab=“positive affect”

41. ti=“positive emotions” or ab=“positive emotions”

42. ti=“benefit finding” or ab=“benefit finding”

43. ti=flow or it=flow or ab=flow

44. ti=positivity or ab=positivity

45. ti=savoring or ab=savoring

46. ti=savouring or ab=savouring

47. ti=“meaning making” or ab=“meaning making”

48. ti=accomplishment or ab=accomplishment

49. ti=“positive relationships” or ab=“positive relationships”

50. ti=engage* or it=engage* or ab=engage*

51. ti=self-efficacy or it=self-efficacy or ab=self-efficacy

52. ti=selfefficacy or ab=selfefficacy

53. ti=mindful* or it=mindful* or ab=mindful*

54. ti=coping or it=coping or ab=coping

55. ti=strength* or it=strength* or ab=strength*

56. ti=“positive psychotherapy” or ab=“positive psychotherapy”

57. ti=“positive psycholog*” or it=“positive psycholog*” or ab=“positive psycholog*”

58. ti=humor or it=humor or ab=humor

59. ti=humour or ab=humour

60. ti=gratitude or it=gratitude or ab=gratitude

61. ti=“health-related quality of life” or ab=“health-related quality of life”

62. ti= hrqol or ab= hrqol

63. ti=“quality of life” or it=“quality of life” or ab=“quality of life”

64. ti=qol or ab=qol

65. ti=satisfaction or it=satisfaction or ab=satisfaction

66. ti=happiness or it=happiness or ab=happiness

67. ti=happy or ab=happy

68. ti=flourish or ab=flourish

69. ti=promot* or it=promot* or ab=promot*

70. ti=protective or it=protective or ab=protective

71. ti=achievement or it=achievement or ab=achievement

72. ti=self-concept or it=self-concept or ab=self-concept

73. ti=selfconcept or ab=selfconcept

74. ti=optimal or ab=optimal

75. ti=wellness or ab=wellness

76. ti=growth or it=growth or ab=growth

77. ti=probem-solving or it=problem-solving or ab=problem-solving

78. ti=cope or ab=cope

79. ti=spirituality or it=spirituality or ab=spirituality

80. ti=committed-action or ab=committed-action

81. 32 or 33 or 34 or 35 or 36 or 37 or 38 or 39 or 40 or 41 or 42 or 43 or 44 or 45 or 46 or 47 or 48 or 49 or 50 or 51 or 52 or 53 or 54 or 55 or 56 or 57 or 58 or 59 or 60 or 61 or 62 or 63 or 64 or 65 or 66 or 67 or 68 or 69 or 70 or 71 or 72 or 73 or 74 or 75 or 76 or 77 or 78 or 79 or 80

82. (7) and (27 or 31) and (81)

**Search Syntax**

The “blocks” are from the “Positive Psychology and Pain Search Term March 18th 2015” file.

**“ti=”** – title

**“ab=”** – abstract

**“it=”** – index terms

1. **(7) and (27 or 31) and (81)**

(ti=pediatric* or it=pediatric* or ab=pediatric* or ti=paediatric* or ab=paediatric* or ti=adolesc* or it=adolesc* or ab=adolesc* or ti=child* or it=child* or ab=child* or ti=infan* or it=infan* or ab=infan* or ti=youth or it=youth or ab=youth) AND (ti=pain or it=pain or ab=pain or ti=headache or it=headache or ab=headache or ti=migraine or it=migraine or ab=migraine or ti="stomach ache" or ab="stomach ache" or ti=stomachache or ab=stomachache or ti="tummy ache" or ab=tummyache or ab=bellyache or ab=earache or ab="ear ache" or ab="tooth ache" or ti=toothache or ab=toothache or ti=odontalgi* or ab=odontalgi* or ti=dysmenorr* or it=dysmenorr* or ab=dysmenorr* or ti=neuralgi* or it=neuralgi* or ab=neuralgi* or ti=fibromyalgia or it=fibromyalgia or ab=fibromyalgia or ti=arthritis or it=arthritis or ab=arthritis or ti="sickle cell" or it="sickle cell" or ab="sickle cell" or ab=sicklecell or ti="quantitative sensory testing" or ab="quantitative sensory testing" or ti=needle* or it=needle* or ab=needle* or ti="cold pressor" or ab="cold pressor") AND (ti=adapt* or it=adapt* or ab=adapt* or ti=resilien* or it=resilien* or ab=resilien* or ti=wellbeing or ab=wellbeing or ti=well-being or it=well-being or ab=well-being or ti=optimis* or it=optimis* or ab=optimis* or ti=hope or it=hope or ab=hope or ti=acceptance or it=acceptance or ab=acceptance or ti="psychological flexibility" or ab="psychological flexibility" or ti="positive affect" or ab="positive affect" or ti="positive emotions" or ab="positive emotions" or ti="benefit finding" or ab="benefit finding" or ti=flow or it=flow or ab=flow or ti=positivity or ab=positivity or ti=savoring or ab=savoring or ti=savouring or ab=savouring or ti="meaning making" or ab="meaning making" or ti=accomplishment or ab=accomplishment or ti="positive relationships" or ab="positive relationships" or ti=engage* or it=engage* or ab=engage* or ti=self-efficacy or it=self-efficacy or ab=self-efficacy or ti=selfefficacy or ab=selfefficacy or ti=mindful* or it=mindful* or ab=mindful* or ti=coping or it=coping or ab=coping or ti=strength* or it=strength* or ab=strength* or ti="positive psychotherapy" or ab="positive psychotherapy" or ti="positive psycholog*" or it="positive psycholog*" or ab="positive psycholog*" or ti=humor or it=humor or ab=humor or ti=humour or ab=humour or ti=gratitude or it=gratitude or ab=gratitude or ti="health-related quality of life" or ab="health-related quality of life" or ti= hrqol or ab= hrqol or ti="quality of life" or it="quality of life" or ab="quality of life" or ti=qol or ab=qol or ti=satisfaction or it=satisfaction or ab=satisfaction or ti=happiness or it=happiness or ab=happiness or ti=happy or ab=happy or ti=flourish or ab=flourish or ti=promot* or it=promot* or ab=promot* or ti=protective or it=protective or ab=protective or ti=achievement or it=achievement or ab=achievement or ti=self-concept or it=self-concept or ab=self-concept or ti=selfconcept or ab=selfconcept or ti=optimal or ab=optimal or ti=wellness or ab=wellness or ti=growth or it=growth or ab=growth or ti=probem-solving or it=problem-solving or ab=problem-solving or ti=cope or ab=cope or ti=spirituality or it=spirituality or ab=spirituality or ti=committed-action or ab=committed-action)

**PubMed**

1. pediatric*[tiab] or pediatric*[mh]

2. paediatric*[tiab]

3. adolesc*[mh] or adolesc*[tiab]

4. child*[tiab] or child*[mh]

5. infan*[tiab] or infan*[mh]

6. youth[tiab] or youth[mh]

7. 1 or 2 or 3 or 4 or 5 or 6

8. pain[tiab] or pain[mh]

9. headache[tiab]

10. migraine[tiab]

11. stomach ache[tiab]

12. stomachache[tiab]

13. tummy ache[tiab]

14. tummyache[tiab]

15. abdominal ache[tiab]

16. belly ache[tiab]

17. bellyache[tiab]

18. earache[tiab]

19. ear ache[tiab]

20. tooth ache[tiab]

21. toothache[tiab]

22. odontalgi*[tiab]

23. dysmenorr*[tiab]

24. neuralgi*[tiab]

25. fibromyalgia[tiab]

26. arthritis[tiab]

27. sickle cell[tiab]

28. sicklecell[tiab]

29. 8 or 9 or 10 or 11 or 12 or 13 or 14 or 15 or 16 or 17 or 18 or 19 or 20 or 21 or 22 or 23 or 24 or 25 or 26 or 27 or 28

30. quantitative sensory testing[tiab]

31. needle*[tiab] or needle*[mh]

32. cold pressor[tiab]

33. 30 or 31 or 32

34. adapt*[tiab]

35. resilien*[tiab]

36. wellbeing[tiab]

37. well-being[tiab] or well-being[mh]

38. optimis*[tiab]

39. hope[tiab] or hope[mh]

40. acceptance[tiab]

41. psychological flexibility[tiab]

42. positive affect[tiab]

43. positive emotions[tiab]

44. benefit finding[tiab]

45. flow[tiab]

46. positivity[tiab]

47. savoring[tiab]

48. savouring[tiab]

49. meaning making[tiab]

50. accomplishment[tiab]

51. positive relationships[tiab]

52. engage*[tiab]

53. self-efficacy[tiab] or self-efficacy[mh]

54. selfefficacy[tiab]

55. mindful*[tiab] or mindful*[mh]

56. coping[tiab] or coping[mh]

57. strength*[tiab]

58. positive psychotherapy[tiab]

59. positive psycholog*[tiab]

60. humor[tiab]

61. humour[tiab]

62. gratitude[tiab]

63. health-related quality of life[tiab]

64. hrqol[tiab]

65. quality of life[tiab] or quality of life[mh]

66. qol[tiab]

67. satisfaction[tiab] or satisfaction[mh]

68. happiness[tiab] or happiness[mh]

69. happy[tiab] or happy[mh]

70. flourish[tiab]

71. promot*[tiab]

72. protective[tiab]

73. achievement[tiab]

74. self-concept[tiab]

75. selfconcept[tiab]

76. optimal[tiab]

77. wellness[tiab]

78. growth[tiab]

79. problem-solving[tiab]

80. cope[tiab]

81. spirituality[tiab]

82. committed-action[tiab]

83. 34 or 35 or 36 or 37 or 38 or 39 or 40 or 41 or 42 or 43 or 44 or 45 or 46 or 47 or 48 or 49 or 50 or 51 or 52 or 53 or 54 or 55 or 56 or 57 or 58 or 59 or 60 or 61 or 62 or 63 or 64 or 65 or 66 or 67 or 68 or 69 or 70 or 71 or 72 or 73 or 74 or 75 or 76 or 77 or 78 or 79 or 80 or 81 or 82

84. (7) and (29 or 33) and (83)

**Search Syntax**

**[tiab]** = Title/Abstract

**[mh]** = MeSH term

1. (**7) and (29 or 33) and (83)**

(((pediatric*[tiab] or pediatric*[mh] or paediatric*[tiab] or adolesc*[mh] or adolesc*[tiab] or child*[tiab] or child*[mh] or infan*[tiab] or infan*[mh] or youth[tiab] or youth[mh])) AND (pain[tiab] or pain[mh] or headache[tiab] or migraine[tiab] or stomach ache[tiab] or stomachache[tiab] or tummy ache[tiab] or tummyache[tiab] or abdominal ache[tiab] or belly ache[tiab] or bellyache[tiab] or earache[tiab] or ear ache[tiab] or tooth ache[tiab] or toothache[tiab] or odontalgi*[tiab] or dysmenorr* [tiab] or neuralgi* [tiab] or fibromyalgia[tiab] or arthritis[tiab] or sickle cell[tiab] or sicklecell[tiab] or quantitative sensory testing[tiab] or needle* [tiab] or needle*[mh] or cold pressor[tiab])) AND (adapt*[tiab] or resilien*[tiab] or wellbeing[tiab] or well-being[tiab] or well-being[mh] or optimis*[tiab] or hope[tiab] or hope[mh] or acceptance[tiab] or psychological flexibility[tiab] or positive affect[tiab] or positive emotions[tiab] or benefit finding[tiab] or flow[tiab] or positivity[tiab] or savoring[tiab] or savouring[tiab] or meaning making[tiab] or accomplishment[tiab] or positive relationships[tiab] or engage*[tiab] or self-efficacy[tiab] or self-efficacy[mh] or selfefficacy[tiab] or mindful*[tiab] or mindful*[mh] or coping[tiab] or coping[mh] or strength*[tiab] or positive psychotherapy[tiab] or positive psycholog*[tiab] or humor[tiab] or humour[tiab] or gratitude[tiab] or health-related quality of life[tiab] or hrqol[tiab] or quality of life[tiab] or quality of life[mh] or qol[tiab] or satisfaction[tiab] or satisfaction[mh] or happiness[tiab] or happiness[mh] or happy[tiab] or happy[mh] or flourish[tiab] or promot*[tiab] or protective[tiab] or achievement[tiab] or self-concept[tiab] or selfconcept[tiab] or optimal[tiab] or wellness[tiab] or growth[tiab] or problem-solving[tiab] or cope[tiab] or spirituality[tiab] or committed-action[tiab])

# Supplementary Tables

As shown in Table 1, all studies are denoted by the first author and year of publication and can be found in the references section of the full paper. Each study has been assigned a unique identification (ID) code that corresponds to the data presented in Tables 2-5 below.

Table 1. A list of the included studies in alphabetical order and assigned ID number.

| ID | First Author & Year | ID | First Author & Year | ID | First Author & Year |
| --- | --- | --- | --- | --- | --- |
| 01 | Anie et al., 2002 | 43 | Hocking et al., 2011 | 85 | Reed-Knight et al., 2018 |
| 02 | Armstrong et al., 1993 | 44 | Holden et al., 1994 | 86 | Reid et al., 1997 |
| 03 | Atoui et al., 2015 | 45 | Holden et al., 1998 | 87 | Reid et al., 2005 |
| 04 | Barakat et al., 2002 | 46 | Huygen et al., 2000 | 88 | Russel et al., 2020 |
| 05 | Barakat et al., 2007 | 47 | Hyacinth et al., 2020 | 89 | Sallfors et al., 2003 |
| 06 | Barakat et al., 2008 | 48 | Jones et al., 2016 | 90 | Sanders et al., 1994 |
| 07 | Barakat et al., 2010 | 49 | Jong et al., 2019 | 91 | Sawyer et al., 2005 |
| 08 | Barbarin et al., 1999 | 50 | Kaczynski et al., 2009 | 92 | Schanberg et al., 1996 |
| 09 | Beneitez et al., 2020 | 51 | Kaczynski et al., 2011 | 93 | Schanberg et al., 1997 |
| 10 | Broome et al., 2001 | 52 | Kaczynski et al., 2013 | 94 | Schatz et al., 2015 |
| 11 | Brown et al., 1993 | 53 | Kaminsky et al., 2006 | 95 | Schlenz et al., 2016 |
| 12 | Bung et al., 2017 | 54 | Kashikar-Zuck et al., 2001 | 96 | Schwartz et al., 2009 |
| 13 | Casey et al., 2000 | 55 | Kashikar-Zuck et al., 2002 | 97 | Seid et al., 2014 |
| 14 | Claar & Scharff, 2007 | 56 | Kashikar- Zuck et al., 2005 | 98 | Sharpe et al., 1994 |
| 15 | Claar & Simons, 2011 | 57 | Kashikar-Zuck et al., 2013 | 99 | Shirkey et al., 2011 |
| 16 | Claar et al., 2008 | 58 | Keogh et al., 2006 | 100 | Sil et al., 2020 |
| 17 | Compas et al., 2006 | 59 | Kliewer & Lewis 1995 | 101 | Simons et al., 2008 |
| 18 | Connelly et al., 2019 | 60 | Kuttner et al., 2006 | 102 | Simon et al., 2009 |
| 19 | Cotton et al., 2009 | 61 | Levy et al., 2010 | 103 | Skogvold & Magnussen, 2019 |
| 20 | Cotton et al. 2012 | 62 | Levy et al., 2017 | 104 | Stinson et al., 2008 |
| 21 | Easterlin et al., 2020 | 63 | Lewis & Kliewer 1996 | 105 | Stinson et al., 2014 |
| 22 | Eccleston et al., 2004 | 64 | Libby & Glenwick 2010 | 106 | Stone et al., 2016 |
| 23 | Fletcher, 2000 | 65 | Lim et al., 2019 | 107 | Thastum et al., 2005 |
| 24 | Forrester et al., 2015 | 66 | Logan et al., 2012 | 108 | Thompson et al., 1993 |
| 25 | Fraga et al., 2019 | 67 | Logan et al., 2013 | 109 | Thompson et al., 1994 |
| 26 | Frare et al., 2002 | 68 | Ludwig et al., 2018 | 110 | Thompson et al., 1999 |
| 27 | Fuggle et al., 1996 | 69 | Lutz et al., 2004 | 111 | Thomsen et al., 2002 |
| 28 | Gil et al., 1993 | 70 | Major et al., 2017 | 112 | van der Veek et al., 2019 |
| 29 | Gil et al., 2001 | 71 | McClellan et al., 2009 | 113 | van Tilburg et al., 2015 |
| 30 | Gilbert, 1995 | 72 | McCormick et al., 2010 | 114 | Voerman et al., 2015 |
| 31 | Gilbert, 1999 | 73 | Mehta, 1992 | 115 | Walker et al., 2005 |
| 32 | Gladstein et al., 1996 | 74 | Meldrum et al., 2009 | 116 | Walker et al., 2007 |
| 33 | Gold et al., 2008 | 75 | Merljin et al., 2003 | 117 | Walker et al., 2008 |
| 34 | Goldenberg et al., 2013 | 76 | Merljin et al., 2006 | 118 | Warschburger et al., 2014 |
| 35 | Graves & Jacob, 2014 | 77 | Mitchell et al., 2007 | 119 | Wassom et al., 2013 |
| 36 | Griffiths & Martin, 1996 | 78 | Nieto et al., 2020 | 120 | Westendorp et al., 2017 |
| 37 | Guell, 2007 | 79 | Nørgaard et al., 2017 | 121 | Wicksell et al., 2005 |
| 38 | Harris et al., 1991 | 80 | Oliver-Carpenter et al., 2011 | 122 | Wojtowicz et al., 2015 |
| 39 | Hechler et al., 2010 | 81 | Oliveria et al., 2017 | 123 | Woods et al., 2019 |
| 40 | Heimann et al., 2018 | 82 | Orr et al., 2017 | 124 | Yetwin et al., 2018 |
| 41 | Hermann et al., 2007 | 83 | Powers et al., 2002 | 125 | Ziadni et al., 2011 |
| 42 | Hildenbrand et al., 2015 | 84 | Prussien et al. 2018 |  |  |

Table 2. The study/participant and methodological characteristics of each included study organized by measure and alphabetically within each measure.

|  | Study & Participant Characteristics | | | | | | | | |  | Methodological Characteristics | |
| --- | --- | --- | --- | --- | --- | --- | --- | --- | --- | --- | --- | --- |
| ^a^ID | ^b^Measures | Country | Chronic pain (*n*) | Non-chronic pain (*n)* | % Female | Target age range (years) | Mean age (SD) | Pain Conditions | Ethnicity/Race |  | Study design | Data type |
| **Pain Coping Questionnaire (PCQ; *n* = 28 studies)** | | | | | | | | | | | | |
| 18 | PCQ | USA | 289 | NA | 72.3% | 12-18 | 14.6 (1.8) | JIA | *Race:* 86% White 14% Other *Ethnicity*: 86.5% Not Hispanic or Latino, 13.5% Hispanic or Latino |  | RCT | Quantitative |
| 22 | PCQ | UK | 80 | NA | 71.3% | 11-17 | 14.5 (1.6) | Multiple | NR |  | Cross-Sectional | Quantitative |
| 34 | PCQ | USA | 1 | NA | 0% | 17 | NA | Multiple | 100% African American |  | Case-study | Quantitative |
| 35 | PCQ | USA | 66 | NA | 53% | 10-17 | 13.7 (1.0) | SCD | 95.5% African American, 4.5% Hispanic/ Caucasian |  | Cross-Sectional | Quantitative |
| 47 | PCQ | USA | 48 | NA | 45.8% | 10-17 | 13.4 (1.9) | SCD | NR |  | Cross-Sectional | Quantitative |
| 48 | PCQ | USA | 60 | NA | 80% | 8-20 | 16.1 (2.5) | Systemic lupus erythematosus | 50% Caucasian, 41% African American, 9% other |  | Cross-Sectional | Quantitative |
| 49 | PCQ Dutch translation | Netherlands | 131 | NA | 77.1% | 9-18 | 13.3 (2.5) | Headache | NR |  | Longitudinal | Quantitative |
| 54 | PCQ | USA | 73 | NA | 78.1% | NR | 14.8 (3.0) | Multiple | 90.4% Caucasian, 6.9% Black, 2.7% Latino |  | Cross-Sectional | Quantitative |
| 55 | PCQ | USA | 36 | NA | 100% | 9-19 | 15.3 (2.3) | Multiple | 94.4% Caucasian, 5.6% Black |  | Cross-Sectional | Quantitative |
| 56 | PCQ | USA | 30 | NA | 100% | 13-17 | 15.8 (1.3) | Fibromyaliga | 93% Caucasian, 7% African American |  | Longitudinal | Quantitative |
| 57 | PCQ | USA | 100 | NA | 93.0% | 11-18 | 15.0 (1.8) | Fibromyalgia | 90% Caucasian, 6% African American, 2% American Indian/Alaskan Native, 1% Asian, 1% Biracial |  | RCT | Quantitative |
| 58 | PCQ | UK | 161 | NA | 71.4% | 11-19 | 14.8 (1.7) | Multiple | NR |  | Cross-Sectional | Quantitative |
| 60 | PCQ | Canada | 25 | NA | 80.0% | 11-18 | 14.2 (2.0) | Abdominal | 88% Caucasian, 12% Other |  | RCT | Quantitative |
| 65 | PCQ | USA | 104 | NA | 53.0% | 10-18 | 12.9 (3.1) | SCD | 96.2% African American. 1% Hispanic/Latino, 1% Asian, 2.8% Unknown |  | Cross-Sectional | Quantitative |
| 68 | PCQ | USA | 100 | NA | 61.0% | 8-18 | 13.5 (2.8) | SCD | 96% African American, 2% Multiracial, 2% Hispanic, 2% Unknown |  | Cross-Sectional | Quantitative |
| 72 | PCQ | USA | 24 | NA | 100% | 11-17 | 14.6 (1.9) | Abdominal | 91.5% Caucasian, 8.5% African American |  | Non-RCT | Quantitative |
| 75 | PCQ | Netherlands | 222 | 148 | 71.6% | 12-18 | 14.9 | Multiple | NR |  | Cross-Sectional | Quantitative |
| 76 | PCQ Dutch translation | Netherlands | 194 | NA | 74.7% | 12-18 | 14.7 (1.4) | Multiple | NR |  | Cross-Sectional | Quantitative |
| 79 | PCQ Danish translation | Denmark | 61 | NA | 60.7% | 10-16 | NR | JA | NR |  | Cross-Sectional | Quantitative |
| 82 | PCQ | Canada | 85 | NA | 68.2% | 10-18 | 999 | Headache | NR |  | Cross-Sectional | Quantitative |
| 86 | PCQ | Canada | 30 | 15 | 86.7% | 11-18 | 14.6 (1.9) | Multiple | NR |  | Cross-Sectional | Quantitative |
| 87 | PCQ | Canada | 30 | 15 | 86.7% | 8-18 | 14.6 (1.9) | Multiple | NR |  | Cross-Sectional | Quantitative |
| 88 | PCQ | USA | 36 | NA | NR | 9-19 | 14.8 (2.2) | NA | 93% Caucasian, 4% African American, 1.5% Asian, 1.5% Hispanic |  | NA | NA |
| 100 | PCQ | USA | 101 | NA | 54.5% | 6-18 | 13.4 (2.9) | SCD | 98.0% Black/African American |  | Longitudinal | Quantitative |
| 104 | PCQ | Canada | 76 | NA | 77.6% | 9-17 | 13.4 (2.5) | JIA | NR |  | Longitudinal | Quantitative |
| 107 | PCQ Danish translation | Denmark | 56 | NA | 80.4% | 7-15 | 11.4 (2.1) | JIA | NR |  | Cross-Sectional | Quantitative |
| 114 | PCQ | The Netherlands | 69 | NA | 76.8% | 12-17 | 14.9 (1.1) | Multiple | NR |  | RCT | Quantitative |
| 121 | PCQ | Sweden | 1 | NA | 100% | 14 | 14 | JIA | NR |  | Case-study | Quantitative |
| **Coping Strategies Questionnaire for Sickle Cell Disease (CSQ-SCD; *n* = 21 studies)** | | | | | | | | | | | | |
| 01 | CSQ | UK | 67 | NA | 44.8% | 7-15 | 11.3 (2.2) | SCD | 61% African, 34% Caribbean, 5% Mediterranean |  | Cross-Sectional | Quantitative |
| 05 | CSQ | USA | 41 | NA | 48.8% | 12-18 | 15.1 (1.8) | SCD | 87.8% African American, 12.7% Other |  | Longitudinal | Quantitative |
| 06 | CSQ | USA | 53 | NA | 58.5% | 12-18 | 14.1 (1.8) | SCD | 94.2% African American, 1.9% White, 3.8% Other |  | Cross-Sectional | Quantitative |
| 07 | CSQ | USA | 37 | NA | 59.5% | 12-18 | 14.2 (1.8) | SCD | 97% African American |  | RCT | Quantitative |
| 12 | CSQ | Germany | 77 | 72 | 100% | 12-17 | 14.8 (1.1) | Headache | NR |  | Cross-Sectional | Quantitative |
| 23 | CSQ | USA | 68 | NA | 52.9% | 13-19 | 16.0 (2.0) | SCD | 100% African American |  | Cross-Sectional | Quantitative |
| 28 | CSQ | USA | 70 | NA | 47.1% | 7-18 | 11.8 (3.4) | SCD | 100% African American |  | Longitudinal | Quantitative |
| 29 | CSQ  Daily Pain Diary | USA | 46 | NA | 52.2% | 8-17 | 12 | SCD | 100% African America |  | RCT | Quantitative |
| 64 | CSQ | USA | 57 | NA | 93.0% | 10-18 | 15.5 (2.0) | Fibromyalgia | 79% white, 9% Hispanic, 3.5% African American, 3.5% Asian American, 5% Unknown |  | Cross-Sectional | Quantitative |
| 77 | CSQ | USA | 48 | NA | NR | 7-13 | 10.7 | SCD | 97.9% African American |  | Cross-Sectional | Quantitative |
| 80 | CSQ | USA | 47 | NA | 48.9% | 6-18 | 11.93 | SCD | 99.1% African American |  | Cross-Sectional | Quantitative |
| 92 | CSQ | USA | 16 | NA | 81.3% | 10-20 | 15 (2.6) | Fibromyalgia | 87.5% Caucasian, 6.2% African American, 6.2% Other |  | Cross-Sectional | Quantitative |
| 93 | CSQ | USA | 56 | NA | 64.3% | 6-20 | 12.4 (3.5) | JCA | 95% Caucasian, 5% African American |  | Cross-Sectional | Quantitative |
| 94 | CSQ | USA | 46 | NA | 58.7% | 8-21 | 13.0 (2.5) | SCD | 93% Black/African American, Non-Hispanic, 4.3% African, 2.2% Biracial |  | RCT | Quantitative |
| 95 | CSQ | USA | 76 | NA | 42.1% | 8-19 | 14.1 (3.2) | SCD | 100% African America  Note: A portion of participants identified as having multiple racial identities. |  | Cross-Sectional | Quantitative |
| 96 | CSQ | USA | 40 | NA | 62.5% | 12-18 | 14 (1.6) | SCD | 97.5% were African American |  | Cross-Sectional | Quantitative |
| 102 | CSQ | USA | 44 | 15 | 64.7% | 12-19 | 15.0 (1.9) | SCD | 90.3% African American |  | Cross-Sectional | Quantitative |
| 108 | CSQ | USA | 50 | NA | 44.0% | 7-12 | 9.0 (1.7) | SCD | Not provided |  | Cross-Sectional | Quantitative |
| 109 | CSQ | USA | 35 | NA | 40.0% | 7-14 | 10.9 (2.2) | SCD | Not provided |  | Longitudinal | Quantitative |
| 110 | CSQ | USA | 109 | NA | 48.6% | 7-17 | 10.8 (3.0) | Multiple | NR |  | Longitudinal | Quantitative |
| 125 | CSQ | USA | 44 | NA | 47.7% | 12-18 | 15 (1.9) | SCD | 87% African American, 2% Caucasian, 11% Other |  | Cross-Sectional | Quantitative |
| **Pain Response Inventory (PRI; *n* = 19 studies)** | | | | | | | | | | | | |
| 15 | PRI | USA | 85 | NA | 85.9% | 13-17 | 15.0 (1.3) | Multiple | 91.9% Caucasian |  | Longitudinal | Quantitative |
| 16 | PRI | USA | 254 | NA | 76.8% | 12-17 | 14.7 (1.5) | Multiple | 90.7% Caucasian |  | Cross-Sectional | Quantitative |
| 50 | PRI | USA | 266 | NA | 66.5% | 8-17 | 13.3 (2.6) | Multiple | 89% Caucasian |  | Cross-Sectional | Quantitative |
| 51 | PRI | USA | 280 | NA | 75.7% | 12-17 | 14.8 (1.5) | Multiple | 91.1% Caucasian |  | Cross-Sectional | Quantitative |
| 52 | PRI | USA | 262 | NA | 68.3% | 11-17 | 14.7 (1.6) | Headache | 89.6% Caucasian |  | Cross-Sectional | Quantitative |
| 53 | PRI | Canada | 50 | NA | 82.0% | 8-17 | 11 (2.8) | Abdominal | 69% Caucasian, 4% Indo-Asian |  | Cross-Sectional | Quantitative |
| 61 | PRI | USA | 200 | NA | 71.5% | 7-17 | 11.2 (2.6) | Abdominal | 96.9% Caucasian |  | RCT | Quantitative |
| 62 | PRI | USA | 316 | NA | 64.6% | 7-12 | 9.4 (1.7) | Abdominal | 77.8% Caucasian, 14.9% Mixed race, 9.8% Hispanic, 4.7% Black/African American, 1.3% American Indian/Alaska Native, 1.3% Asian |  | Longitudinal | Quantitative |
| 66 | PRI | USA | 157 | NA | 86.6% | 10-18 | 14.7 (2.1) | Multiple | 98% Caucasian |  | Longitudinal | Quantitative |
| 67 | PRI | USA | 614 | NA | 72.5% | 7-18 | 13.9 (0.2) | CRPS | 88.3% Caucasian, 11.7% Other |  | Cross-Sectional | Quantitative |
| 85 | PRI | USA | 183 | NA | 47.5% | 8-18 | 13.8 (2.7) | Abdominal | 4.4% Hispanic, 88% Caucasian |  | Cross-Sectional | Quantitative |
| 99 | PRI  Daily Diary Interview | USA | 116 | NA | 63.8% | 8-15 | 10.8 (2.1) | Multiple | 93.0% Caucasian |  | Longitudinal | Quantitative |
| 101 | PRI | USA | 217 | NA | 77.4% | 12-17 | 14.8 (1.5) | Multiple | 92.5% Caucasian |  | Cross-Sectional | Quantitative |
| 106 | PRI | USA | 871 | NA | 59.2% | 8-18 | 11.6 (2.5) | Abdominal | 87.8% Caucasian, 4.1% African American, 0.7% Hispanic, 0.7% Asian, 1.6% Other, 5.1% Did not report race |  | Longitudinal | Quantitative |
| 112 | PRI – Dutch Version | The Netherlands | 117 | NA | 69.2% | 7-18 | 11.9 (2.8) | Abdominal | NR |  | Cross-Sectional | Quantitative |
| 113 | PRI | USA | 389 | NA | 61.2% | 7-18 | 12.5 (2.7) | Abdominal | 92% Caucasian, 4.5% Hispanic |  | Cross-Sectional | Quantitative |
| 115 | PRI  Daily Dairy Interview | USA | 133 | NA | 57.1% | 8-15 | 10.8 (2.1) | Abdominal | 95% Caucasian |  | Cross-Sectional | Quantitative |
| 117 | PRI | USA | 699 | NA | 59.2% | 8-18 | 11.6 (2.5) | Abdominal | 93% Caucasian |  | Cross-Sectional | Quantitative |
| 119 | PRI | USA | 20 | NA | 80.0% | 13-17 | 15.2 (1.1) | Abdominal | 86% Caucasian |  | RCT | Quantitative |
| **Pediatric Pain Coping Inventory (PPCI; *n* = 7 studies)** | | | | | | | | | | | | |
| 25 | PPCI -Portuguese translation | Brazil | 100 | 50 | 84% | 8-18 | 12.9 (2.8) | Multiple | NR |  | Cross-Sectional | Quantitative |
| 39 | PPCI-R - German version | Germany | 167 | NA | 61.7% | 11-18 | NR | Multiple | NR |  | Longitudinal | Quantitative |
| 40 | PPCI-R - German version | Germany | 60 | NA | 57.0% | 8-18 | 14.4 (2.0) | Other: Somatic symptom and related disorders | NR |  | Longitudinal | Quantitative |
| 91 | PPCI | Australia | 54 | NA | 57.4% | 8-18 | 12.8 (3.3) | JIA | NR |  | Cross-Sectional | Quantitative |
| 97 | PPCI | USA | 230 | NA | 69.1% | 2-16 | 9.4 (4.5) | JIA | 92.6% white |  | Cross-Sectional | Quantitative |
| 118 | PPCI-R - German version | Germany | 170 | NA | 64.1% | 8-18 | 11.8 (2.4) | Abdominal | NR |  | Cross-Sectional | Quantitative |
| 123 | PPCI | USA | 114 | NA | 50.0% | 6-19 | NR | Headache | NR |  | Cross-Sectional | Quantitative |
| **KidCope (*n* = 6 studies)** | | | | | | | | | | | | |
| 04 | KIDCOPE | USA | 81 | NA | 42.0% | 8-18 | 7.5 (5.5) | SCD | 88.8% African American, 1.2% Latino, 10% Other |  | Cross-Sectional | Quantitative |
| 32 | KIDCOPE | USA | 37 | NA | 78.4% | 7-17 | 13 | Headache | 49% were white |  | Cross-Sectional | Quantitative |
| 33 | KIDCOPE | USA | 41 | 97 | 39% | 7-17 | 11.13 (2.6) | SCD | 100% African American |  | Cross-Sectional | Quantitative |
| 44 | KIDCOPE | USA | 70 | NA | 57.1% | 6-17 | 12 | Headache | 53% Caucasian, 47% African American |  | Cross-Sectional | Quantitative |
| 45 | KIDCOPE | USA | 57 | NA | 45.6% | 7-12 | 8.9 (1.6) | Headache | 36% African American, 64% European-American |  | Cross-Sectional | Quantitative |
| 69 | KIDCOPE | USA | 81 | NA | 42.0% | 8-18 | 7.5 (5.5) | SCD | 88.8% African American, 1.3% Latino, 10% Other |  | Cross-Sectional | Quantitative |
| **Responses to Stress Questionnaire (*n* = 5 studies)** | | | | | | | | | | | | |
| 17 | RSQ – Abdominal version | USA | 164 | NA | 54.9% | 11-18 | 13.7 (2.0) | Abdominal | 94% Caucasian |  | Cross-Sectional | Quantitative |
| 43 | RSQ | USA | 44 | NA | 68.2% | 8-16 | 11.77 (7.2) | Abdominal | 81.8% Caucasian, 16% African Americans, 2.2% Native American |  | Cross-Sectional | Quantitative |
| 84 | RSQ – SCD version | USA | 44 | NA | 43.2% | 6-16 | 9.3 (3.1) | SCD | 97.7% African American |  | Cross-Sectional | Quantitative |
| 111 | RSQ –Abdominal version | USA | 174 | NA | 66.7% | 7-18 | 11.6 (2.9) | Abdominal | 94% Caucasian |  | Cross-Sectional | Quantitative |
| 124 | RSQ | USA | 65 | NA | 76.9% | 8-18 | 13.6 (2.6) | Multiple | 55.4% Caucasian, 23.1% Latina, 6.2% African American, 1.5% Asian/Pacific Islander, 13.8% Other |  | Cross-Sectional | Quantitative |
| **Coping Strategies Inventory (CSI; *n* = 4 studies)** | | | | | | | | | | | | |
| 02 | CSI | USA | 52 | 26 | 46.2% | 7-17 | 10.3 (2.9) | SCD | 87.2% African America, 11.5% Haitian, 1.9% Hispanic Black |  | Cross-Sectional | Quantitative |
| 11 | CSI | USA | 61 | 15 | 51.3% | 6-17 | 8.8 (4.0) | SCD | NR |  | Cross-Sectional | Quantitative |
| 13 | CSI | USA | 118 | NA | 40.7% | 5-18 | 10.0 (3.0) | SCD | NR |  | Cross-Sectional | Quantitative |
| 98 | CSI | USA | 55 | NA | 47.3% | 3-16 | 8.7 (4.0) | SCD | 100% African American |  | Cross-Sectional | Quantitative |
| **Other questionnaires (*n* = 11 studies)** | | | | | | | | | | | | |
| 10 | A-COPE  SCSI | USA | 97 | NA | 54.6% | 6-18.5 | 12.3 (1.8) | SCD | 100% African American |  | RCT | Quantitative |
| 14 | PPQ | USA | 297 | NA | 70.7% | 8-17 | 13.8 (2.4) | Multiple | 92.6% Caucasian |  | Cross-Sectional | Qualitative |
| 19 | Brief R-COPE  Semi-structured interview | USA | 48 | NA | 52.1% | 11-19 | 14.8 (2.0) | SCD | 98.5% African American |  | Cross-Sectional | Quantitative |
| 36 | CHAS | Australia | 42 | NA | 50% | 10-12 | 11.3 (0.6) | Headache | NR |  | RCT | Quantitative |
| 38 | Child and Adolescent Coping Inventory | USA | 12 | 12 | 58.3% | 6-11 | NR | JRA | NR |  | Cross-Sectional | Quantitative |
| 41 | PRCQ-C | Germany | 148 | 253 | 59.6% | 7-18 | 10.77 (1.9) | Multiple | NR |  | Longitudinal | Quantitative |
| 42 | HICUPS | USA | 15 | NA | 53.3% | 6-14 | 9.13 (3.0) | SCD | 100% African American |  | Cross-Sectional | Qualitative |
| 59 | CCSC | USA | 39 | NA | 64.1% | 7-16 | 11.0 (2.9) | SCD | 100% African American |  | Cross-Sectional | Quantitative |
| 63 | CCSC | USA | 39 | NA | 64.1% | 7-16 | 11 (2.9) | SCD | NR |  | Cross-Sectional | Quantitative |
| 70 | Ways of Coping Inventory | Hungary | 8 | NA | 87.5% | 10-19 | 14.8 (2.5) | Multiple | NR |  | Longitudinal | Quantitative |
| 120 | UCL | The Netherlands | 172 | NA | 85.5% | 10-20 | 16.2 (2.5) | Multiple | NR |  | Longitudinal | Quantitative |
| **Other measures/methods (*n* = 24)** | | | | | | | | | | | | |
| 03 | Semi-structured interview | Lebanon | 12 | NA | 58.3% | 12-17 | NR | SCD | 50% Lebanese, 50% Palestinian |  | Cross-Sectional | Qualitative |
| 08 | Sick Role Adoption Index | USA | 182 | NA | 45.1% | 8-17 | NR | SCD | NR |  | Cross-Sectional | Quantitative |
| 09 | Semi-structured interview | Spain | 14 | NA | 78.6% | 11-18 | NR | Other (JIA) | NR |  | Cross-Sectional | Qualitative |
| 20 | Drawings | USA | 19 | NA | 57.9% | 5-10 | 8.1 (1.8) | SCD | 100% African American |  | Cross-Sectional | Qualitative |
| 21 | Semi-structured interview | USA | 18 | NA | 55.6% | 8-18 | 13.0 | Abdominal | 89% White, 6% Hispanic, 6% Asian |  | Cross-Sectional | Qualitative |
| 24 | Semi-structured adolescent interview | Jamaica | 6 | NA | NR | 18-19 | NR | SCD | NR |  | Cross-Sectional | Qualitative |
| 26 | Eco-cultural Family Interview | Italy | 48 | NA | 70.8% | 8-14 | 11.0 (1.9) | Headache | NR |  | Cross-Sectional | Quantitative |
| 27 | Children's Health Diary | UK | 25 | 25 | 68.0% | 6-16 | 9.67 (3.2) | SCD | 60% African American, 40% Afro-Caribbean |  | Longitudinal | Quantitative |
| 30 | Interview  Medical records and clinic notes | USA | 8 | NA | NR | 6-12 | NR | Headache | NR |  | Cross-Sectional | Mixed |
| 31 | Interview  Medical records and clinic notes | USA | 8 | NA | 33.3% | 6-12 | NR | Headache | 87.5% Caucasian 12.5% Hispanic |  | Cross-Sectional | Mixed-Method |
| 37 | Observations  Unstructured interviews | UK, Germany | 4 | NR | 75% | 7-16 | NR | JIA | NR |  | Ethnography | Qualitative |
| 46 | Unclear | Netherlands | 47 | 52 | 64.6% | 7-16 | 12 (3.2) | JCA | NR |  | Cross-Sectional | Quantitative |
| 71 | DPAD | USA | 19 | NA | 68.4% | 9-20 | 13.4 (2.9) | SCD | 100% African American |  | Longitudinal | Quantitative |
| 73 | Parent observations and child ratings of “coping strategies” on a scale of 0 to 10 | India | 18 | NA | 38.9% | Unclear | 10.7 | Headache | 100% Indian |  | Longitudinal | Quantitative |
| 74 | Semi-structured interview | USA | 45 | NA | 71.1% | 10-18 | 14.7 (2.4) | Multiple | 64.4% Caucasian, 11.1% Latino, 2.2% African American, 2.2% Asian American/ Pacific Islander, 20.0% Other (mixed race) |  | Cross-Sectional | Qualitative |
| 78 | Written Narrative Task | Spain | 39 | NA | 53.8% | 9-17 | 12.2 (1.9) | Abdominal | NR |  | Cross-Sectional | Qualitative |
| 81 | AEH-DOR | Brazil | 7 | NA | 42.9% | 8-10 | 999 | SCD | NR |  | Longitudinal | Quantitative |
| 83 | DPAD | USA | 3 | NA | 33.3% | 9-12 | 10.7 (2.1) | SCD | 100% African America |  | Case studies/ longitudinal | Quantitative |
| 89 | Question: “What do you do to cope with your pain?” | Sweden | 125 | NA | 65.5% | 10-17 | 14.1 | JCA | NR |  | Cross-Sectional | Qualitative |
| 90 | Self-reported ratings of using specific coping strategies | Australia | 44 | NA | NR | 7-14 | 9.2 (2.0) | Abdominal | NR |  | RCT | Quantitative |
| 103 | Semi-structured interview | Norway | 17 | NA | 65.0% | 14-19 | NR | Headache | NR |  | Cross-Sectional | Qualitative |
| 105 | Semi-structured interview | Canada | 23 | NA | 77.3% | 14-18 | NR | Multiple | NR |  | Cross-Sectional | Qualitative |
| 116 | Daily Diary Interview  Structured interview | USA | 143 | 104 | 56.7% | 8-15 | 10.8 (2.0) | Abdominal | 95% Caucasian |  | Cross-Sectional | Quantitative |
| 122 | Clinician ratings | USA | 100 | NA | 79.0% | 13-19 | 15.8 (2.7) | Multiple | NR |  | Retrospective chart review | Mixed-Method |

NR = not reported; Measures: A-COPE= Adolescent Coping Orientation for Problem Experiences; AEH-DOR= Computerized Assessment Instrument of Coping with Hospitalization Pain; CSI = Coping Strategies Inventory; CSQ = Coping Strategies Questionnaire; CCSC= Children’s Coping Strategies Checklist; CHAS= Children’s Headache Assessment Scale; DDI= Daily Diary Interview; DPAD= Daily Pain and Activity Diary; HICUPS= How I Coped Under Pressure Scale; PCQ= Pain Coping Questionnaire; PPCI= Paediatric Pain Coping Inventory; PPQ= Paediatric Pain Questionnaire; PRI= Pain Response Questionnaire; PRCQ= Pain Related Cognitions for Questionnaire for Children; RSQ= Response to Stress Questionnaire; SCSI= Schoolagers Coping Strategies Inventory; UCL= Utrecht Coping List. Pain Conditions: SCD = sickle cell disease; CRPS= complex regional pain syndrome; JA= juvenile arthritis; JCA= juvenile chronic arthritis; JIA= juvenile idiopathic arthritis; JRA= juvenile rheumatoid arthritis.

^a^Studies are indicated by a identification number corresponding to Table 1.

^b^This table is organized by measure. Some studies (IDs: 19, 29, 99, 115) included both a questionnaire and another type of measure (e.g., daily diary, interview). These studies were sorted by the questionnaire employed.

Table 3. A list of studies (*n* = 11) that were deemed to assess proactive coping responses (i.e., efforts or goals undertaken in advance of a painful episode to prevent it or reduce its severity) and a description of the measure used.

| ^a^Studies | Measure Type | Description/Sample Questions |
| --- | --- | --- |
| 08 | Sick Role Adoption Index | “The sick role adoption index assesses a particular strategy for coping with SCD characterized by adoption of the sick role. The index reveals the extent to which the ill child is perceived as using the illness as an excuse to avoid age-appropriate responsibilities, acts as though the illness were a problem on a day-to-day basis, and acts as though giving in to the illness rather than taking it in stride.” (p. 363) |
| 14 | Paediatric Pain Questionnaire (PPQ) – select items from the PPQ were adapted and asked to parents of youth with chronic pain in an interview format. | “Assuming that the pain continues, what kinds of things do you think you/your child should do now that will help him/her later on?” (p. 289) |
| 20 | Semi-structured interview and drawings | “The interviews were primarily comprised of questions related to general and religious/spiritual coping. For example, interviewers asked children to ‘‘Draw a picture of the things in your life that help you deal with being sick’’ (general coping). Children were then asked about the use of prayer and God’s feelings about them having SCD and were asked to ‘‘Draw a picture of you and God’’ (or other preferred term for a Higher Power) ‘‘when you are sick/in pain’’ (religious/spiritual coping). Probes and follow-up questions were used as necessary to explore the child’s responses.” (p. 245) |
| 37 | Ethnographic observations and unstructured interviews | “Participant observation is the prime method for conducting anthropological ethnographic research, and analyzing illness narratives as recounted by the patients can give in-depth insights into lived experiences (Kleinman, 1988). It demands that the researcher spend time with the people under study, participating in their daily activities and exploring them from the participant’s perspective” (p. 886) |
| 103 | Semi-structured interview | “A semistructured interview guide was used to ensure that key topics would be identified. Open‐ended questions made it possible for the adolescents to talk openly about their experiences of living and dealing with chronic headaches in their daily lives” (p. 2) |
| 29, 71, 83, 99, 115, 116 | Daily diary-recorded measures | Interviews, questionnaires, or mobile applications used to track youth’s use of coping responses on a daily basis. |

^a^Studies are indicated by a identification number corresponding to Table 1

Table 4. A list of 168 coping responses measured, analyzed and reported on across the included studies presented in alphabetical order.

| No. | Coping Response | ^a^Studies |
| --- | --- | --- |
| 1 | Acceptance | 03, 16, 66, 81, 117, 119 |
| 2 | Accommodative coping | 50, 51, 66, 99, 101, 106, 112, 115, 116, 119 |
| 3 | Active coping | 01, 04, 31, 42, 50, 51, 52, 53, 59, 63, 66, 69, 74, 90, 99, 101, 106, 112, 113, 115, 119 |
| 4 | Adaptive copers | 15, 30 |
| 5 | Adaptive coping | 30,60 |
| 6 | Adherence coping | 125 |
| 7 | Affective coping | 01, 30, 31 |
| 8 | Aggression | 38 |
| 9 | Angry-destructive coping | 12 |
| 10 | Anticipatory coping | 30, 31 |
| 11 | Anxiety | 38 |
| 12 | Approach coping | 18, 33, 35, 42, 47, 54, 55, 56, 57, 58, 60, 65, 68, 71,7 5, 76, 82, 86, 87, 104, 114, 121 |
| 13 | Aspirations to cope | 46 |
| 14 | Attention-gaining strategies | 90 |
| 15 | Avoidant actions | 42 |
| 16 | Avoiding problems | 10 |
| 17 | Avoidance coping | 12, 20, 33, 42, 45, 59, 63, 81, 89, 120 |
| 18 | Avoidant copers | 16, 15, 117 |
| 19 | Behavioural coping/strategies | 21 |
| 20 | Behavioural disengagement | 53, 113,117 |
| 21 | Behavioural distraction | 21, 54, 55, 57, 58, 72, 79, 107, 114 |
| 22 | Being humorous | 10 |
| 23 | Blaming others | 44, 45, 79, 81 |
| 24 | Blaming self | 44 |
| 25 | Body awareness | 74 |
| 26 | Breathing exercises/Deep breathing | 21,71 |
| 27 | Calming self-statements | 92 |
| 28 | Cognitive decision-making | 42 |
| 29 | Cognitive avoidance | 42 |
| 30 | Cognitive coping | 21, 30, 31, 61 |
| 31 | Cognitive distraction | 22, 54, 55, 57, 58, 61, 72, 79, 92, 107, 114 |
| 32 | Cognitive refocusing | 11, 21, 91, 97 |
| 33 | Cognitive restructuring | 42, 45, 81 |
| 34 | Cognitive self-instruction | 25, 31, 40, 91, 97, 123, |
| 35 | Cognitive strategy | 89 |
| 36 | Cognitive-behavioural coping | 30, 31 |
| 37 | Comfort seeking | 81 |
| 38 | Complaints | 81, 90 |
| 39 | Complaining to mother | 90 |
| 40 | Compliance with treatment | 89 |
| 41 | Condition-specific strategies | 117 |
| 42 | Confidence in medical care | 21 |
| 43 | Controlling strategy | 89 |
| 44 | Copers | 30, 31 |
| 45 | Coping attempts | 05, 06, 07, 28, 29, 64, 54, 77,80, 83, 95, 102, 108, 109, 110 |
| 46 | Coping responses | 32, 36, 44, 61 |
| 47 | Deep breathing | 21, 71 |
| 48 | Deferring religious/spiritual coping style | 19 |
| 49 | Denial | 03, 38 |
| 50 | Dependent copers | 15, 16, 117 |
| 51 | Different feeling | 44 |
| 52 | Disengagement coping | 02, 11, 13, 16, 17, 43, 84, 98, 111, 124 |
| 53 | Distracting actions | 62 |
| 54 | Distraction | 10, 16, 23, 24, 25, 44, 45, 55, 56, 59, 60, 63, 72, 81, 82, 103, 104, 117, 123 |
| 55 | Diverting attention | 92 |
| 56 | Divine intervention | 03 |
| 57 | Doctor/hospital visits | 27 |
| 58 | Drawing | 10 |
| 59 | Emotion-focused avoidance | 18, 35, 47, 54, 55, 56, 58, 60, 65, 68, 75, 76, 82, 86, 87, 104, 114 |
| 60 | Emotion-focused coping | 61, 62, 72, 121 |
| 61 | Emotion-focused disengagement | 02, 11 |
| 62 | Emotion-focused engagement | 02, 11 |
| 63 | Emotion-focused support | 42 |
| 64 | Emotional regulation | 45, 81 |
| 65 | Engaged copers | 15, 16, 117 |
| 66 | Engagement coping | 02, 11, 13, 98 |
| 67 | Engaging in a demanding activity | 10 |
| 68 | Expression of emotion | 11, 42, 44 |
| 69 | Externalizing | 22, 54, 55, 58, 79, 82, 107, 114 |
| 70 | Focusing on treatment benefits | 21 |
| 71 | Getting on with things | 74 |
| 72 | Helplessness/become helpless | 25, 44, 81,123, |
| 73 | Humour | 3, 10, 30 |
| 74 | Ill child coping style | 8 |
| 75 | I'll never surrender coping | 30, 31 |
| 76 | Ignore/Ignoring pain/Ignoring pain sensations | 16, 23, 61, 92, 93 |
| 77 | Illness-focused strategy | 29, 35 |
| 78 | Imagery | 30, 71 |
| 79 | Immaturity | 38 |
| 80 | Inconsistent copers | 15, 16, 117 |
| 81 | Increasing behavioural activity level | 23, 92, 93 |
| 82 | Information seeking | 22, 55, 54, 58, 79, 81, 114 |
| 83 | Infrequent copers | 15, 16, 117 |
| 84 | Internalizing | 5, 22, 42, 52, 54, 58, 79, 82, 114 |
| 85 | Investing in close friends | 10 |
| 86 | Low-level activity and walking | 103 |
| 87 | Maintaining a future orientation | 44, 45 |
| 88 | Maintaining optimism | 21 |
| 89 | Making plans and setting routines | 21, 42, 103 |
| 90 | Maladaptive coper | 15, 30 |
| 91 | Massage and guard | 113, 117 |
| 92 | Medical coping | 2, 11, 27, 90, 98, 103 |
| 93 | Minimizing pain | 3, 16, 61, 62, 117 |
| 94 | Negative actions | 42 |
| 95 | Negative coping | 45 |
| 96 | Negative religious coping | 19, 20 |
| 97 | Negative thinking | 5, 28, 64, 77, 80, 83, 94, 95, 96, 102, 108, 109, 110 |
| 99 | Negotiation | 81 |
| 100 | Normalcy | 3, 42 |
| 101 | Optimism | 10 |
| 102 | Passive adherence | 1, 5, 28, 77, 80, 83, 94, 102, 108, 109, 110 |
| 103 | Passive coping | 4, 39, 40, 50, 51, 52, 53, 61, 66, 67, 69, 74, 85, 90, 99, 101, 106, 112, 113, 116, 119, 122 |
| 104 | Passive disengagement | 16 |
| 105 | Passive problem-solving | 118 |
| 106 | Passive reaction | 120 |
| 107 | Perpetual alterations | 45 |
| 108 | Personal strategies | 78 |
| 109 | Perspective coping | 30, 31 |
| 110 | Pharmacological strategies | 105 |
| 111 | Physical strategies | 105 |
| 112 | Physiological | 38 |
| 113 | Planning ahead | 74 |
| 114 | Positive coping self-statements/Positive self-talk | 16, 22, 23, 39, 41, 54, 55, 57, 58, 72, 79, 90, 92, 93, 107, 117 |
| 115 | Positive religious coping | 19, 20 |
| 116 | Positive thinking | 44 |
| 117 | Practical disease management | 42 |
| 118 | Pray and hope | 23, 92 |
| 119 | Preventative coping | 30, 31 |
| 120 | Primary control coping | 17, 84, 111 |
| 121 | Primary control engagement coping | 43, 111, 124 |
| 122 | Problem-focused avoidance | 11, 35, 47, 58, 65, 68, 69, 72, 75, 76, 82, 86, 87, 114, 121 |
| 123 | Problem-focused coping | 30, 31, 41, 61, 62 |
| 124 | Problem-focused Disengagement | 2, 11 |
| 125 | Problem-focused Engagement | 2, 11 |
| 126 | Problem-focused support | 42 |
| 127 | Problem-solving/Problem-solve | 10, 11, 12, 16, 22, 25, 41, 44, 45, 54, 55, 57, 58, 73, 79, 113, 114, 117, 123 |
| 128 | Problem-solving self-efficacy | 91, 97 |
| 129 | Professional support seeking | 10, 78, 112 |
| 130 | Progressive muscle relaxation | 71 |
| 131 | Rational thinking coping | 92 |
| 132 | Recovery strategy | 89 |
| 133 | Reinterpreting pain sensations | 23, 92, 93 |
| 134 | Relaxation (Try to relax, stay relaxed, relaxing) | 10 |
| 135 | Religious/spiritual coping | 10, 19, 20, 24, 30, 31, 42, 93 |
| 136 | Requesting medication | 90 |
| 137 | Resignation | 30, 31, 45 |
| 138 | Rest/resting | 90 |
| 139 | Routines in daily life | 103 |
| 140 | Rumination | 81 |
| 141 | Secondary control coping | 84, 124 |
| 142 | Secondary control engagement coping | 17, 43, 111 |
| 143 | Seeking diversions | 10 |
| 144 | Seeking medical help | 42, 45 |
| 145 | Seeking professional support | 10, 78, 112 |
| 146 | Seeking spiritual support | 10, 19, 20, 24, 30, 31, 42, 93 |
| 147 | Seeking understanding | 42 |
| 148 | Self-criticism | 11, 45 |
| 149 | Self-hurt | 38 |
| 150 | Self-improvement | 38 |
| 151 | Self-isolation | 16, 53, 116, 117 |
| 152 | Self-reliance | 10 |
| 153 | Self-reliant copers | 16, 15, 117 |
| 154 | Sick role adoption | 8 |
| 155 | Sleep | 10 |
| 156 | Social support (seeking social support) | 3, 9, 10, 11, 12, 16, 21, 22, 25, 27, 30, 34, 38, 39, 40, 42, 44, 45, 54, 55, 58, 59, 63, 72, 78, 79, 89, 91, 97, 105, 107, 113, 114, 116, 117, 118, 123 |
| 157 | Social withdrawal | 11, 30, 45, 81 |
| 158 | Solving family problems | 10, 11, 12, 16, 22, 25, 41, 44, 45, 54, 55, 57, 58, 73, 79, 113, 114, 117, 123 |
| 159 | Stoicism | 117 |
| 160 | Striving for rest and being alone | 91, 97, 103 |
| 161 | Taking control | 21, 42 |
| 162 | Taking it in stride | 8 |
| 163 | Think about it | 10 |
| 164 | Thought stopping | 30 |
| 165 | Use of a comfort item | 42 |
| 166 | Venting feelings | 10 |
| 167 | Wishful thinking | 11, 44, 45 |
| 168 | Withdrawn | 38, 44 |

^a^Studies are indicated by a reference number corresponding to Table 1.

Table 5. A list of discrepancies noted among studies rated ‘low’ on measurement and/or conceptual consistency for the five most frequently used questionnaires.

| Measure | ^a^Measurement Discrepancies | ^a^Conceptual Discrepancies |
| --- | --- | --- |
| Pain Coping Questionnaire (PCQ) | 1) Included participants beyond the age-range for which the scale was developed.   - A sample of youth that extends one year above or below the age-range for which the PCQ assessed (*n* = 9; IDs: 18, 57, 60, 65, 68, 75, 76, 82, 100). - A sample of youth that extends two or more years above or below the age-range for which the PCQ was assessed (*n* = 3; IDs: 48, 55, 58).   2) Used alternate composite scores.   - Items pertaining to the subscale ‘positive self-statements’ were scored as ‘problem-focused avoidance’ instead of ‘approach’ (*n* = 3; IDs: 75, 76, 121). - A composite score made up of ‘problem-solving’, ‘positive self-statements’, ‘behavioural distraction’ and ‘cognitive distraction’ was computed (*n* = 1; ID: 57). - Computed the total score using all subscales of the PCQ (*n* = 1; ID: 48). - Three items from the PCQ were used to measure pain self-efficacy (*n* = 1; ID: 100).   3) One or more subscales were missing (*n* = 2; IDs: 76, 107).  4) Response options were coded as 0-4 instead of 1-5 (*n* = 2; IDs: 60, 68).  5) Subscales were computed by taking the sum of the item-level responses instead of the mean (*n* = 1; ID: 100). | 1) Used the term ‘positive approach’ instead of ‘approach coping’ (*n* = 1; ID: 47).  2) Used the term ‘distraction’ instead of ‘problem-focused avoidance’ (*n* = 4; IDs: 55, 57, 60, 104).  3) ‘Positive self-statements’ were conceptualized as a type of ‘problem-focused avoidance’ instead of ‘approach’ coping (*n* = 3; IDs: 75, 76, 121). Based on the original scale development study for the PCQ, there was evidence to suggest that the positive self-statements load onto both higher-order domains in a community sample of youth; however, these results were not supported in a subsequent sample of youth with chronic pain as it loaded onto approach coping only. As a result, it was concluded by the scale developers that while positive self-statements may enable the use of distraction strategies (problem-focused avoidance), they better reflect an effort to deal with pain directly (approach). |
| Coping Strategies Questionnaire for Sickle Cell Disease (CSQ-SCD) | 1) Included participants beyond the age-range for which the scale was developed.   - A sample of youth that extends one year above or below the age-range for which the CSQ-SCD was assessed (*n* = 7; IDs: 05, 06, 07, 28, 64, 80, 96). - A sample of youth that extends two or more years above or below the age-range for which the CSQ-SCD was assessed (*n* = 6; IDs: 92, 93, 94, 95, 102, 125).   2) A sample of youth with a pain condition other than SCD (*n* = 2; IDs: 64 = fibromyalgia, 92 = juvenile primary fibromyalgia syndrome).  3) Used alternate composite scores (total coping attempts, rational thinking coping) (*n* = 3; IDs: 64, 92, 93).  4) Did not administer or score the following subscales in their computation of higher-order factor scores: resting, taking fluids, heat/cold, fear self-statement, anger self-statements, isolation (*n* = 1; ID: 64).  5) Used a 6- instead of 7-point Likert scale (*n* = 2; IDs: 77, 80).  6) Used a 44-item version of the CSQ-SCD (*n* = 2; IDs: 92, 93).  7) Subscales were computed by taking the sum of the item-level responses instead of the mean (*n* = 1; IDs: 06, 94). | 1) Alternate terminology was used for the coping responses assessed.   - Used the term ‘illness-focused strategy’ instead of ‘passive avoidance’ (*n* = 1; ID: 29). - Used the term ‘active coping’ instead of ‘coping attempts’ (*n* = 1; ID: 01) - Used the term ‘affective coping’ instead of ‘negative thinking’ (*n* = 1; ID: 01). - Used the term ‘adherence’ instead of ‘passive adherence’ (*n* = 1; ID: 125). - Used the term ‘calming self-statements’ instead of ‘coping self-statements’ (*n* = 1; ID: 93).   2) Inconsistent categorization of primary coping responses into secondary factors (i.e., grouping ‘pray/hope’ under ‘coping attempts’; described ‘negative thinking’ using 'catastrophizing’ only) (*n* = 1; ID: 64).  3) Inconsistent descriptor for ‘coping attempts’:   - Described as CBT strategies (as opposed to specific cognitive and behavioural strategies) with examples of CBT strategies that are not captured by the measure itself such as relaxation and attention, imagery, distraction) (*n* = 1; ID: 94). - Described by its purported relationship with adaptive outcomes as opposed to focusing on the nature of the coping responses (i.e., active coping responses including cognitive and behavioural strategies) (*n* = 1; ID: 95). |
| Pain Response Inventory (PRI) | 1) Included participants aged one year above or below the age-range for which the PRI was assessed (*n* = 3; IDs: 61, 62, 66).  2) A sample of youth with pain conditions other than abdominal pain (*n* = 2; IDs: 50, 101).  3) Missing one or more subscales (typically ‘rest’, ‘stoicism’, ‘condition-specific’, and/or ‘massage/guard’) (*n* = 4; IDs: 50, 51, 101, 113).  4) Subscales were computed by taking the sum the item-level responses instead of the mean (*n* = 1; ID: 67).  5) Used the subscale ‘distraction’ in the computation of ‘active coping’ instead of ‘accommodative coping’ (*n* = 1; ID: 50). | 1) Missing subscales or the inconsistent categorization of lower-order subscales into higher-order subscales:   - Did not include the subscale ‘stoicism’ in the conceptualization of ‘accommodative’ coping (*n* = 3; IDs: 50, 66, 101). - Missing subscales or incorrect subscales used to describe active coping (*n* = 3; IDs: 50, 51, 113).   2) Used the term ‘activity restriction’ instead of ‘behaviour disengagement’ (*n* = 1; ID: 52).  3) Distraction was conceptualized as a type of ‘active coping’ instead of ‘accommodative coping’ (*n* = 1; ID: 50). |
| Pediatric Pain Coping Inventory (PPCI) | 1) Included participants aged two or more years above or below the age-range for which the PPCI was assessed (*n* = 2; IDs: 91, 97).  2) Included a sample of youth with a pain condition other than musculoskeletal pain (*n* = 1; ID: 97). | 1) The original version of the scale was used rather than the finalized version that was recommended after the original testing (i.e., measured ‘distraction’, ‘problem-solving’ and ‘catastrophizing/-helplessness’ instead of ‘strive for rest and be alone’, ‘cognitive refocusing’ and ‘problem-solving self-efficacy’) (*n* = 1; ID: 123). |
| KidCope | 1) Included participants aged two or more years above or below the age-range for which the KidCope was assessed (*n* = 5; IDs: 04, 33, 44, 45, 69).  2) Used higher-order factors that were not proposed by the original scale development study (*n* = 3; IDs: 04, 69 = Active vs. Passive, 33 = Negative/Avoidance vs. Positive/Approach).  3) Used a 4-point Likert scale for the adolescent version instead of a ‘yes/no’ response (*n* = 1; ID: 44). | 1) Used alternate composite scores:   - ‘Active’ vs ‘passive’ coping (*n* = 2; IDs: 04, 69) - ‘Negative/avoidance’ and ‘positive/approach’ (*n* = 1; ID: 33).   2) Mis-labelled subscales/the inclusion of different coping responses (e.g., used the term ‘withdrawal’ instead ‘social withdrawal’; added remain positive, blame self instead of self-criticism, express emotions, relax, become helpless, different feeling, focus on future, taking medication/laying down) (*n* = 1; ID: 44). |

Note: The intention of this table is to highlight the types of inconsistencies noted in the measurement and conceptualizations of coping. However, it is important to note that a ‘discrepancy’ does not necessary mean that the authors used the scale incorrectly or inadequately. Instead, a discrepancy highlights a difference that impacts our ability to make direct comparisons across studies.

^a^Studies are indicated by an identification number corresponding to Table 1.

# Sample Overview Document

A sample overview document is provided below. Overview documents were used to extract relevant information from the scale development studies corresponding to the questionnaires identified in this review.

Pain Coping Questionnaire

**Citation:** Reid GJ, Gilbert CA, McGrath PJ. The pain coping questionnaire: preliminary validation. Pain. 1998 May 1;76(1-2):83-96.

| **Purpose of Measure** | A child and adolescent measure focused on assessing specific types of coping strategies. |
| --- | --- |
| **Theoretical Background and Scale Development** | The higher-order factors of the PCQ incorporate two conceptual models of coping: approach/avoidance and problem-/emotion-focused conceptualizations.   - Approach: directed towards the stressor - Avoidance: directed away from the stressor - Problem-focused: direct attempts to deal with stressor - Emotion-focused: direct attempts to regulate and express emotional reactions to the stressor (Note: The PCQ Emotion-focused avoidance scale attempts to focus only on unregulated, freely expressed emotional reactions to avoid confounding emotional regulation and expression. Seeking social support and positive self-statements were selected as active attempts to regulate emotional reaction.)   **Citation:** Roth S, Cohen LJ. Approach, avoidance, and coping with stress. American psychologist. 1986 Jul;41(7):813. |
| **Population measure was validated for** | **Study 1:**   - Community school sample (*N* = 340, Age range = Grades 3 to 12, *Mean age*= 12.7, *SD* = 2.67, 219 females, 114 males, 7 unreported gender).   **Study 2:**   - Children with arthritis and their parents (*N*= 28, Age range = 7-17, *Mean age*= 12.8, *SD* = 2.50, 22 females, 6 males). - Children with headaches recruited from a Neurology clinic and parents (*N* = 48, Age range = 7-17,*Mean age* =12.0, *SD* =2.65, 26 females, 22 males). |
| **Structure and Classification of Coping Constructs (i.e., subscales)**    ***include descriptors and internal reliability; ND = no descriptor** | Consists of 8 lower-order subscales representing coping strategies nested within 3 higher-order factors.  1. Approach (alpha = .89) – measure direct attempts to deal with the pain and the use of active methods to regulate feelings when in the pain   1. information seeking (.79) - ND 2. problem solving (.86) - ND 3. seeking social support (.86) - ND 4. positive self-statements (.82) - ND   2. Problem focused avoidance (.86) – measures attempt to disengage from the pain   1. behavioral distraction (.78) - ND 2. cognitive distraction (.85) - ND   3. Emotion-focused avoidance (.85) – measures strategies in which emotions are freely expressed and strategies that reflect a lack of effort to regulate feelings when in pain   1. externalizing (.81) - ND 2. internalizing/catastrophizing (.82) - ND |
| **Number of Items** | 39 |
| **Response Options** | Indicated how often (1 = never, 2 = hardly ever, 3 = sometimes, 4 = often, 5 = very often) they used each of 39 coping strategies. |
| **Proactive vs. Reactive** | Reactive: ‘When I am hurt or in pain for a few hours or days, I...’ |
| **Situational vs. General** | Situational (pain) |
| **Self- vs. Parent-Report** | Self- and parent-report |
| **Scoring** | Subscales were scored by averaging the items that loaded on each factor. |
| **Additional Notes** | - Based a preliminary research with the PCQ in community sample of youth (Study 1), there is some evidence to suggest the positive self-statements loads onto both the ‘problem-focused avoidance’ and ‘approach’ factors; however, these results were not supported in youth with chronic pain (Study 2). The authors concluded that ‘positive self-statements’ may enable the use of distraction strategies, but better reflect an effort to deal with pain directly. Given these results and corresponding conclusions, the results of Study 2 were used to develop this overview document. |
